# Fast and accurate resolution of ecDNA sequence using Cycle-Extractor

**DOI:** 10.64898/2026.03.10.710955

**Published:** 2026-04-15

**Authors:** Mahsa Faizrahnemoon, Jens Luebeck, King L. Hung, Suhas Rao, Gino Prasad, Ivy Tsz-Lo Wong, Matthew G. Jones, Paul S. Mischel, Howard Y. Chang, Kaiyuan Zhu, Vineet Bafna

**Affiliations:** 1 Department of Computer Science & Engineering, UC San Diego, La Jolla, CA, USA; 2 Department of Neuroscience, Scripps Research, La Jolla, CA, USA; 3 Department of Biomedical Informatics, Harvard Medical School, Boston, MA, USA; 4 Department of Pathology, Stanford University School of Medicine, Stanford, CA, USA; 5 Department of Biology, Massachusetts Institute of Technology, Cambridge, MA, USA; 6 Koch Institute for Integrative Cancer Research, Massachusetts Institute of Technology, Cambridge, MA, USA; 7 Institute for Medical Engineering and Science, Massachusetts Institute of Technology, Cambridge, MA, USA; 8 Sarafan ChEM-H, Stanford University, Stanford, CA, USA; 9 Amgen Research, South San Francisco, CA, USA; 10 School of Computer Science, Shanghai Jiao Tong University, Shanghai, China; 11 Halicioğlu Data Science Institute, UC San Diego, La Jolla, CA, USA

**Keywords:** extrachromosomal DNA (ecDNA), cancer genomics, breakpoint graphs, cycle reconstruction, mixed-integer linear programming (MILP), long-read and short-read sequencing

## Abstract

Extrachromosomal DNA (ecDNA) plays a key role in cancer pathology. EcDNAs mediate high oncogene amplification and expression and worse patient outcomes. Accurately determining the structure of these circular molecules is essential for understanding their function, yet reconstructing ecDNA cycles from sequencing data remains challenging. We introduce Cycle-Extractor (CE) for reconstruction. CE accepts a breakpoint graph derived from either short or long read sequencing data as input and extracts a cycle with the maximum length-weighted-copy-number. CE utilizes a mixed-integer linear program (MILP) and a separate traversal procedure, enabling fast optimization and compatibility with free solvers.

We evaluated CE against CoRAL (long-read-based quadratic optimization), Decoil (long-reads), and AmpliconArchitect (AA for short reads) on both simulated data and real cancer cell lines. On simulated ecDNA, CE achieves performance comparable to CoRAL across three accuracy metrics and consistently outperforms AA and Decoil. On cancer cell lines, CE produces longer and heavier cycles than AA, and achieves performance similar to CoRAL. Moreover, CE is, on average, 40× faster than CoRAL. These results demonstrate that CE accurately reconstructs ecDNA from both short- and long-read sequencing data, while long-read inputs allow CE to recover more complete and higher-confidence ecDNA structures. CE improved the prediction of many ecDNA structures. On a PC3 ecDNA containing *MYC*, CE uses ONT data to reconstruct a substantially larger and higher-copy sequence (4.2 Mbp) compared to the short-read-derived reconstruction (690 Kbp). CRISPR-CATCH experiments confirm the presence of a large ecDNA molecule, validating the long-read-based CE reconstruction.

## 1 Introduction

Extrachromosomal DNA (ecDNA) is the most common source of amplified oncogenes across human cancers^1–4^. Studies on large cohorts have revealed that ecDNAs are present in approximately 14%-17% of tumors across multiple cancer types, with particularly high prevalence in glioblastoma and other aggressive malignancies^4,5^. These circular DNA molecules, typically ranging from kilobases to megabases in size^4^, drive tumor formation^6^, evolution^1,7–9^ and therapeutic resistance^10–12^. Unlike chromosomal amplifications, ecDNAs lack centromeres and segregate asymmetrically during cell division^13,14^, leading to rapid changes in copy numbers and intratumoral heterogeneity^8^. The unique properties of ecDNAs, including their ability to amplify oncogenes^4,5^, enhance chromatin accessibility^15^, and facilitate long-range regulatory interactions^16^, together make them powerful mediators of tumor adaptation and progression.

EcDNAs are not the only circular DNA species in cells. The similarly named extrachromosomal, circular DNA (eccDNA) are smaller circles (1–50 Kbp) found at low copy number in all cells^17^. The vast majority (97%) of eccDNA are smaller than 25Kbp, formed by simple circularization, and do not contain intact and active genes. EccDNA exploration tools focus on the question of presence/absence and quantity (how many molecules can be detected)^17–24^. In contrast, we focus here on ecDNAs, which are are much larger, often multi-chromosomal, carry enhancers and intact genes, and are found exclusively in cancer cells^5^.

To harness the clinical potential of ecDNA, two distinct, albeit related capabilities are needed: *detection* and *reconstruction* of ecDNA sequences. Determining whether or not the tumor genome has ecDNA can provide prognostic information for treatment. Clinical trials are underway for drugs that specifically treat patients with ecDNA^25^, and detection of ecDNA is required for patient selection. Moreover, large landscape pan-cancer studies correlate ecDNA presence with other phenotypic variables, providing insight into the phenotypic consequence of ecDNA during tumor progression^4,5^. Reconstruction of the ecDNA sequence, on the other hand, is necessary to understand the content of ecDNA. The ecDNA sequence information has been used to identify oncogenes amplified by ecDNA^5^, immunomodulatory genes^4^, ecDNA with only regulatory sequences^4^, ecDNA-ecDNA interaction^26^, enhancer hijacking^27^, and the 3-dimensional structure of ecDNA^28^. We focus here on the reconstruction problem.

Whole genome sequencing (WGS) based approaches have become the standard method for both detection and reconstruction problems. The current computational methods typically follow a parallel strategy for both, starting first with construction of a *breakpoint (or amplicon) graph* that represents amplified genomic regions and their novel adjacencies, using mapped WGS reads^29,30^. For detection, algorithms identify ecDNA by searching for cyclic paths in the graph that exhibit high copy number amplification^5,31^.

Because the graph encodes all breakpoints participating in the ecDNA, the reconstruction problem corresponds to extraction of (potentially non-simple) cycles from the amplicon graph. For reconstruction, these cycles must be accurately extracted from the graph to determine the complete ecDNA sequence and structure. Therefore, unlike diploid genome assembly, ecDNA reconstruction faces the following unique challenges. First, ecDNAs can exhibit high structural complexity, containing many breakpoints that create complex rearrangements^32,33^. Some breakpoints can be missed by current sequencing and mapping technologies, particularly when they overlap with low-complexity or centromeric regions^34–37^. Second, large genomic segments can be duplicated within a single ecDNA molecule, appearing multiple times, with potentially different orientations and contexts, which complicates the determination of the correct order of segments^26,28,35^. Third, ecDNA typically occurs as a heterogeneous population - multiple distinct ecDNA species often coexist within the same tumor^38^. When these species share genomic segments, accurate reconstruction and copy-number estimation become challenging.

Recent developments focus on reconstruction utilizing long-reads (ONT, Pacbio)^35,39^. Long-reads improve cycle extraction by (a) better mapping of all breakpoints, including those that span repetitive or low-complexity regions, and (b) occasionally spanning regions of high multiplicity reducing ambiguities in reconstruction. CoRAL^35^ specifically focused on the problem of extracting cycles that best explain the total copy number and breakpoints in the amplicon graph (see Results), and showed reasonable results on simulations and on cancer cell-lines. However, it uses a mixed integer quadratically constrained programming (MIQCP) that is computationally demanding, and lowers utility for users with no access to MIQCP solvers. Here, we develop Cycle-Extractor(CE), which transforms all quadratic constraints to Mixed Integer Linear Programming (MILP) formulations, improving running time by an order of magnitude while maintaining or exceeding its performance. We tested CE on extensive simulations and on cell-lines, and also tested its ability to detect heterogeneity, where multiple ecDNAs share genomic regions. Finally, by abstracting and separating cycle extraction from graph reconstruction, we could apply CE to better predict ecDNA sequences using only short-read data, increasing its utility for analysis of complex rearrangements.

## 2 Methods

### 2.1 A high-level description of Cycle-Extractor

**Figure 1:**
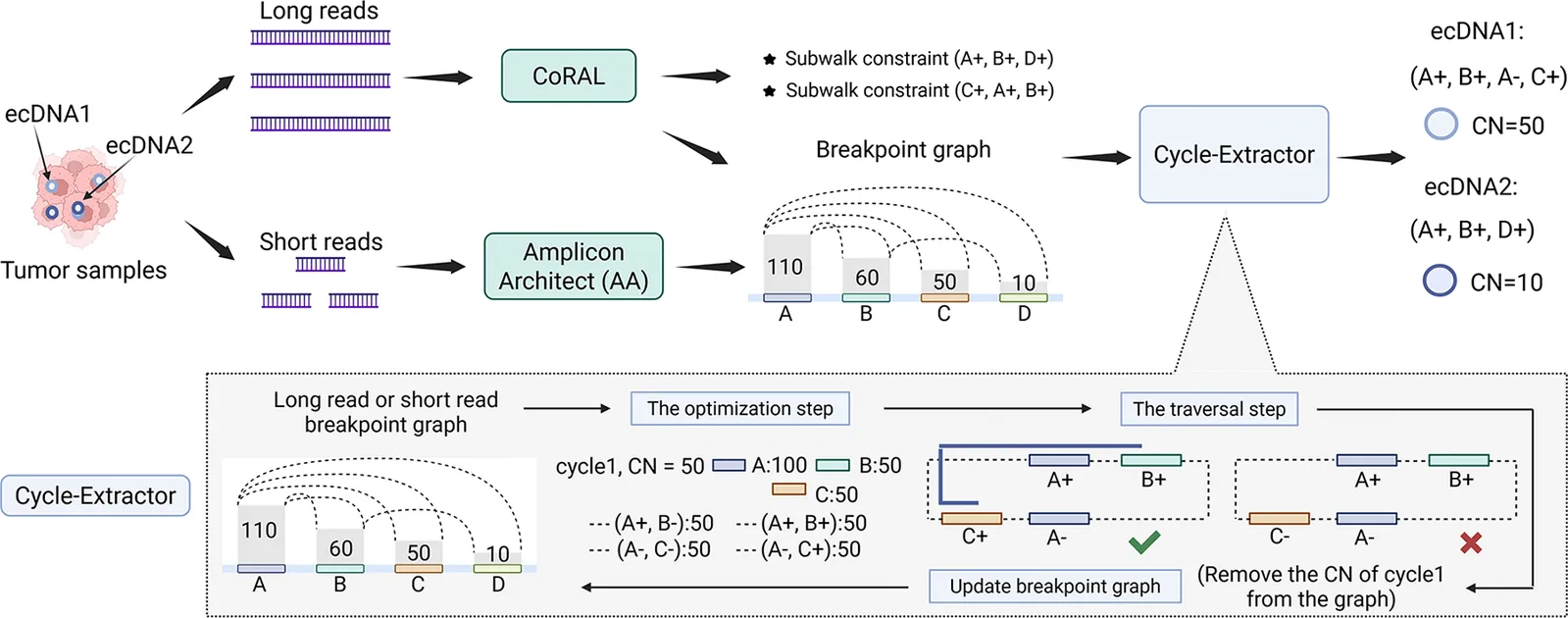
Overview of Cycle-Extractor(CE). CE takes as input, an amplicon graph generated using short-reads (e.g., from AA) or long-reads (e.g., from CoRAL). It applies a mixed-integer linear program to iteratively extract heaviest cycles from the graph (optimization step), and producing ordered traversals of edges (traversal step). From these traversals, CE selects the cycle that satisfies the maximum number of subwalk constraints. The graph is updated by decreasing the copy number of the edges in the selected cycle, and the process is repeated until the amplification is explained.

Cycle-Extractor (CE) takes an *amplicon graph* (or *CN-weighted breakpoint graph*) and a set of *subwalk constraints* as input (Figure 1), both defined below. The (undirected) amplicon graph is described by G=(V,E,C,ℓ), where the edge set $E=E_{s}\cup E_{d}\cup E_{c}$ is the union of *sequence, concordant,* and *discordant* edges. Sequence edges correspond to unbroken chromosomal segments in ecDNA. Discordant edges connect the beginning/end of one sequence edge to itself (indicating *inverted duplication* or *foldback*), or another sequence edge located elsewhere in the genome; while concordant edges connect the end of one sequence edge to the beginning of an adjacent sequence edge on the same chromosome. Each edge (u,v) is associated with a copy-number $C_{uv}$, and length $\ell_{uv}$. The length of the segment edge is the number of nucleotides in the corresponding segment, while ℓ(u,v)=0 for all $(u,v)\in E_{c}\cup E_{d}$.

CE only allows *walks* W in G in which segment edges alternate with discordant or concordant edges. A walk W is cyclic if it begins and ends at the same vertex. A walk W with copy-number $C_{W}$ is *feasible* if the copy number of each edge (u,v) used in the walk satisfies $C_{W}\cdot m_{uv}\leq C_{uv}$, where $m_{uv}$ is the number of times an edge (u,v) is used in W (i.e., its *multiplicity*). The *length-weighted-copy-number* of a walk is defined by $LWCN\left(W,C_{W}\right)=C_{W}\cdot length(W)$. CE optimizes over feasible cyclic walks to output the *heaviest* (maximizing $LWCN\left(W,C_{W}\right)$) cyclic walk and maximize the number of subwalk constraints satisfied. At a high level, CE has two steps. In the first, *optimization* step, it iteratively computes $C_{W}$ and an Eulerian subset of edges (u,v), along with their multiplicities, that comprise the heaviest cyclic walk^1^. Then the copy numbers from these edges are removed from the graph, and the next iteration proceeds until most of the length weighted copy number the graph G (≥α⋅LWCN(G), by default we set α=0.9) is consumed. In the second, *traversal* step, it identifies the actual ordering of the edges in each walk.

One key advantage of long reads is that they provide subwalk constraints that guide reconstruction. Long reads sampled from a cyclic walk W are subwalks of W. However, real samples may exhibit heterogeneity of structure, where multiple ecDNA may have similar but non-identical sequences, and subwalks may be sampled from different cyclic walks. We model subwalks using $p_{uv}^{j}\in \{0,\;1\}$ indicating if edge (u,v) is in subwalk $P_{j}$. We then incorporate constraints corresponding to each subwalk. Given a set of subwalk constraints, 𝒫, CE finds a feasible alternating cycle that simultaneously maximizes LWCN and the number of satisfied subwalk constraints.

### 2.2 The optimization step of CE

The CE MILP utilizes input encodings and a small number of tunable parameters:

M: maximum multiplicity allowed for concordant and discordant edges

$\gamma (W):=0.01\sum_{(u,v)\in W}C_{uv}\ell_{uv}$, and balances weights of subwalk constraints relative to LWCN in the objective.

$p_{uv}^{j}\in \{0,\;1\}$ : indicates if edge (u,v) is in subwalk $p_{j}$

#### Variables.

F∈R: copy number assigned to edges in the solution but with multiplicity one.

$x_{uv}\in \left\{0,\;1\right\}\;:x_{uv}=1$ if edge (u,v) is selected, and 0 otherwise.

$f_{uv}\in R$ : copy number assigned to edge (u,v) in the solution, should be multiples of F.

$y_{uvm}\in \{0,\;1\}$ : (auxiliary) $y_{uvm}=1$ iff (u,v) has multiplicity m∈{1≤m≤M} for each discordant edge in a feasible walk.

$z_{uvm}\in R$ : The auxiliary variable is used to constrain $z_{uvm}=m\cdot F\cdot y_{uvm}$ which is a quadratic formula but is linearized later.

$P_{j}\in \{0,\;1\}$ : indicates if subwalk $p_{j}$ is satisfied 1≤j≤p.

$d_{u}\in \{0,\;1,\;2,\;..\}$ : indicates the order of the nodes, used in connectivity constraints.

$c_{uv}^{+}\in \{0,\;1\}$ : be a binary variable that is 1 if the direction is from u to v, again used in connectivity constraints.

#### CE MILP.

At each iteration, CE maximizes the sum of LWCN and γ-weighted subwalk constraint using

$$max\;\sum_{(u,v)\in E}f_{uv}\ell_{uv}+\gamma \frac{\sum_{j}P_{j}}{\left|𝒫\right|}$$

while satisfying the following constraints.

1. **capacity**: For any edge (u,v) in a feasible solution, its copy number $\left(f_{uv}\right)$ must not exceed its capacity $\left(C_{uv}\right)$. If the edge is not chosen in the solution, its copy number is zero.

$$f_{uv}\leq C_{uv}\cdot x_{uv}\forall (u,v)\in E$$

- **balance**: The sum of the solution copy number of discordant and concordant edges that connect to a node is equal to the solution copy number of the sequence edge connected to that node.

$$\sum_{(w,u)\in E_{c}\cup E_{c}}f_{wu}=\sum_{(u,v)\in E_{s}}f_{uv}$$

- **copy number F with multiplicity one:** Note that if edge (u,v) is picked in the solution (i.e., $x_{uv}=1$), then $F\leq f_{uv}$, but otherwise (u,v) imposes no constraint. For this purpose we introduce a sufficiently large constant $C_{\text{max}}$ (e.g., $C_{\text{max}}=M\cdot {max}_{(u,v)\in E}f_{uv}$).

$$F\leq f_{uv}+C_{max}\cdot \left(1-x_{uv}\right)\forall (u,v)\in E_{c}\cup E_{d}$$

- **discordant edge multiplicity constraint**: The multiplicity of a discordant edge is managed using

$$\sum_{m=1}^{M}y_{uvm}=x_{uv}\;\forall (u,v)\in E_{d}.$$

Subsequently the copy of number of the edge is given by the (quadratic) constraint

$$f_{uv}=\sum_{m=1}^{M}m\cdot F\cdot y_{uvm}=\sum_{m=1}^{M}z_{uvm}\;\forall \left(u,v\right)\in E_{d}$$

which guarantees $f_{uv}$ are multiples of F. Due to the balance condition, we can prove that $f_{uv}$ is multiple of F for each concordant and each sequence edge (Supplementary Methods).

- **subwalk constraints:** If a subwalk is in the solution $\left(P_{j}=1\right)$, all of its edges are in the solution. If a subwalk in not in the solution $\left(P_{j}\neq 0\right)$, there is not constraint here.

$$x_{uv}\geq P_{j}\cdot p_{uv}^{j}\;\forall j\in \left\{1,\ldots ,p\right\}\;\forall \left(u,v\right)\in E\cap p_{j}$$

where $p_{uv}^{j}\in \{0,\;1\}$ is a constant indicating if edge (u,v) is in subwalk $p_{j}$.

Additional constraints including the linearization of discordant edge multiplicity constraint can also be found in Supplementary Methods.

### 2.3 Resolving multiple disconnected cycles

CE maximizes the length-weighted-copy-number, aiming to identify the heaviest solution. With no connectivity requirements, it can generate multiple disconnected cycles within a single iteration, as long as LWCN improves. However, this behavior can result in a reduction of the copy numbers for some cycles. For example, if there are two disjoint cycles with length 100 Kbp each, and CN 20 and 40, respectively, CE could report a ‘single’ solution outputting both cycles with CN 20 (LWCN=4M). To prevent this, we implemented two methods.

In the first, *connectivity-enforcement* method, we added MILP constraints to enforce that exactly one cycle with maximum LWCN is output per iteration (Supplemental Methods). In the previous example, the constraints ensure that only one cycle with CN=40 is output. The second cycle and other cycles are output one at a time in future iterations. In the second, *CN-enhancement* method, we allowed the MILP to output multiple disjoint cycles initially, but subsequently increase the CN of each cycle to the minimum copy number of any edge in that cycle. Among all cycles initially produced, we output the cycle with the maximum enhanced copy number, and reiterate.

In the Results section below, we refer to connectivity-enforcement method as CEc, and the CN-enhancement method as CE, treating it as the default. We tested CE and CEc for accuracy and speed.

### 2.4 The traversal step of CE

The output of the optimization step is a set of edges, (u,v)∈𝒲, and the multiplicities assigned to them, $m_{uv}=\frac{f_{uv}}{F}$. The graph defined by these edges is Eulerian and may admit multiple Eulerian cycles^40^. We perform multiple (default 100) traversals and select a cyclic walk which satisfies the maximum number of subwalk constraints. A modified Hierholzer's algorithm is used to perform the traversal and is applied as follows:

CE maintains a count $r_{uv}$ of the number of times each edge (u,v) remains unused, initialized as its multiplicity $m_{uv}$. It starts with the sequence edge $(u,v)\in E_{s}$ with positive $r_{uv}$ that begins at the canonically smallest chromosome and position, with positive orientation (i.e., starting with the node corresponding to the smaller position). The algorithm then alternates between selecting a non-sequence edge (discordant or concordant) and a sequence edge. When a non-sequence edge is to be chosen, CE randomly selects a discordant or concordant edge (adjacent to the current node) with $r_{uv}>0$ and heads to the next node. Since each node connects to only one sequence edge, the next sequence edge is automatically determined; and if the node corresponds to the larger endpoint in the sequence edge, the orientation is negative, otherwise it is positive. This process continues until the walk returns to its starting node, thereby forming a closed walk. Finally, all closed walks are iteratively merged into a single walk by identifying shared nodes and rotating the edges for the cycle to be inserted.

## 3 Results

### Datasets and metrics.

We simulated 75 distinct cyclic structures using ecSimulator (https://github.com/AmpliconSuite/ecSimulator) closely following CoRAL^35^. Very briefly, each structure contains a collection of between 1 and 20 oriented genomic segments joined end to end. The end of the last segment is joined to the beginning of the first segment to complete the cyclic structure. The simulated structures modeled the known mechanisms of ecDNA formation including episomal^7^, chromothripsis^33,41^, and 2-foldback^42,43^, and were additionally modified using internal structural variations. Next, long and short reads were simulated from the simulated ecDNA sequence at one of three coverages (50X, 100X, or 250X) modeling the increased copy number of ecDNA, and merged with reads from one of five simulated normal, diploid genomes (each with 13X coverage).

From long-read input, an amplicon graph was generated using CoRAL. Cycle reconstruction was performed using CoRAL and Cycle-Extractor for comparisons. For short-reads, the amplicon graph was generated using AA, and cycle extraction was performed using AA and Cycle-Extractor.

We additionally analyzed 31 amplicon graphs from cancer cell-lines (Supp. Table S2), 26 with Illumina WGS, and 12 amplicons with long-reads (ONT) whole genome sequencing, with both sequencing modalities for 7 amplicons. All cell-lines are known to carry ecDNA from prior cytogenetics experiments. For the cell-lines, the true ecDNA sequence is not known, and therefore, we tested them on the previously defined LWCN (length-weighted-copy-number) and LWCNR (ratio) metrics. The tools are all designed for the user to have some control on the stopping criteria. For all experiments below, and all methods (except AA), we stopped when 90% of the total LWCN in the graph was explained, or if the current iteration had not solved the MILP after 2 hours. For AA, such a control is not available, and the default mode was used.

#### CE matches or exceeds CoRAL, Decoil and AmpliconArchitect (AA) performance in simulations.

No single metric captures the quality of reconstructed cycles, and we utilized 3 metrics: First, to measure the nucleotide overlap, consider the true and reconstructed cycles each as multi-sets T, R (including multiplicities) of nucleotide positions. The **Cycle Interval Overlap (CIO)** metric given by

$$CIO(T,R)=\frac{\left|T\;\cap \;R\right|}{\left|T\;\cup \;R\right|},$$

represents the Jaccard index of the shared nucleotides. Next, **Reconstruction Length Error (RLE)** reports the normalized difference in amplicon lengths computed as

$$\left(\frac{\left|L_{t}\;-\;L_{r}\right|}{\left|L_{t}\right|}\right),$$

where $L_{t}$ and $L_{r}$ denote the lengths of the true and reconstructed cycles, respectively. Finally, **Cyclic Longest Common Subsequence (LCS)** measures the length of the longest common subsequence contained in the true cycle and the reconstructed cycle after eliminating intervals that are not found in both, and normalizing to the length of the true cycle.

**Figure 2:**
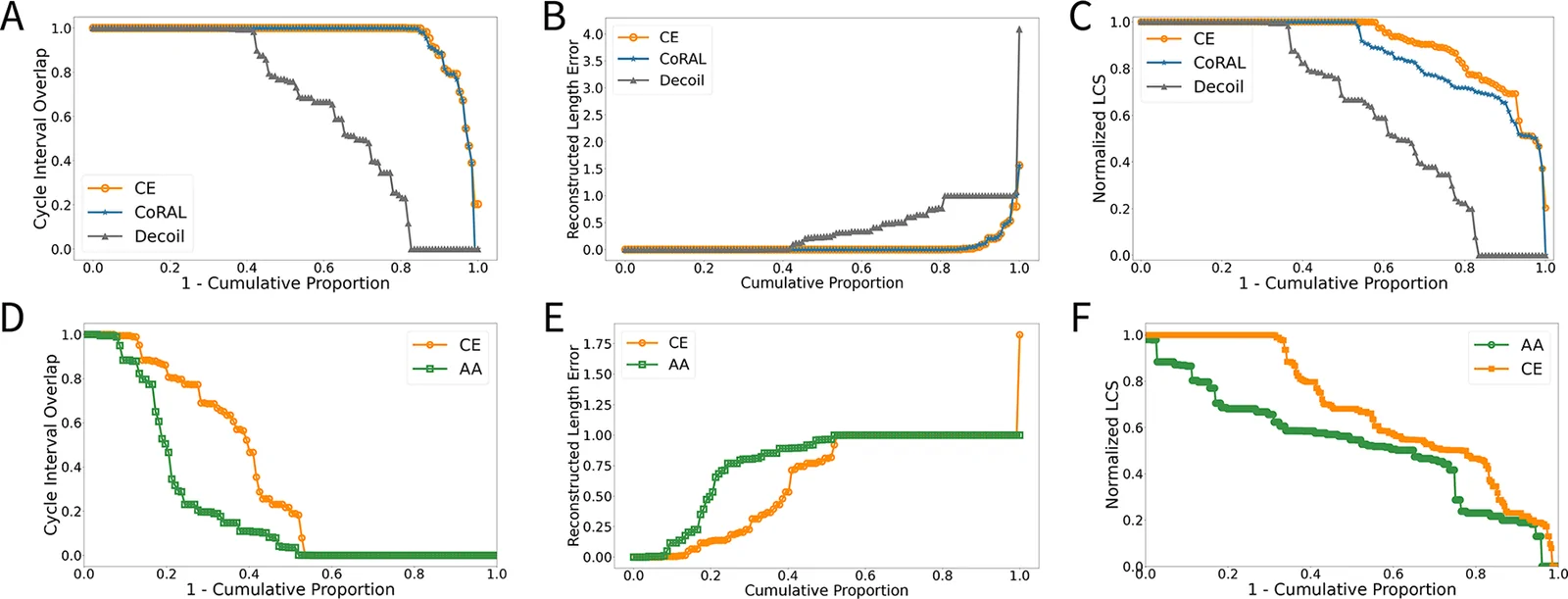
CE accuracy in reconstruction on simulated data. (A-C): CIO, RLE, and LCS metrics comparing CE to CoRAL and Decoil on long-read simulations. For any threshold of a metric the results show the fraction that matched or improved upon the metric. (D-F): Comparison between CE and AA on short-read simulations.

CE performed at least as well as CoRAL (Figure 2A–C) and outperformed Decoil^39^ across all three metrics. For the CIO metric, Decoil was correct about 40% of the time in contrast with CE and CoRAL, which were correct 80% of the time. In about 20% of the samples, Decoil did not return any cycles. Intriguingly, CE improved upon CoRAL for the LCS metric in nearly half of the cases. A partial explanation could be better satisfaction of subwalk constraints, where CE satisfied 7.6% more subwalk constraints than CoRAL. Additionally, CoRAL makes a design choice which gives the same priority to extracting walks and cycles. In some cases where a breakpoint edge is missed, CoRAL selects a walk that is heavier than any available cycle. In summary, the CoRAL and CE reconstructions, while not identical, match up well overall.

The cyclic LCS metric could be dominated by a single long, unbroken segment even when the breakpoint ordering elsewhere was incorrect. To assess if this inflated our LCS metrics, we plotted the distribution of segment lengths and ecDNA sizes (Supplementary Figure S1). A large majority of the fragments were less than 250Kbp, much smaller than the average size of ecDNA (2Mbp), discounting long fragments as impacting the score. Moreover, CE had 100% accuracy in 62% of the cases, which can be achieved only with a perfect reconstruction.

To assess the stability of the traversal step, we performed multiple runs of the randomized Eulerian traversal on the optimized edge sets. We evaluated the consistency of the resulting cycle orderings using the normalized longest common subsequence (LCS) between cycles obtained across runs. The inferred cycles exhibit high agreement across runs (Supplementary Figure S2). Importantly, we did not observe substantially different ecDNA structures across runs. While minor variations in traversal order may arise due to local ambiguities in the Eulerian graph, these differences do not affect the overall structure or the set of constituent edges in the inferred cycles. This stability is expected, as the MILP step significantly constrains the solution space, leaving limited flexibility in the traversal.

For short-reads, CE performed much better than AA on all metrics, showcasing the power of optimization in extracting the heaviest cycle (Figure 2D-F). While the amplicons used to simulate short and long-read data were the same, the long-read data missed fewer breakpoints and also provided subwalk constraints. Therefore, the cumulative performance of CE on short-reads was worse than its own performance on long-reads across all three metrics (Supplementary Figure S3). Manual exploration confirmed that this was due to missed breakpoints in short-read analysis, showcasing the additional sensitivity of long-reads for breakpoint detection. In some simulations, missed breakpoint edges prevented cycle formation (CIO=0 and RLE=1) or shortened the cycle. This happened often with short-reads but sometimes also for long-reads.

Finally, we also tested CE on additional difficult cases where two true cycles sharing genomic segments were simulated. When the two cycles had very similar copy numbers, CE merged them into a longer, heavier cycle (Supplementary Figure S4A). However, when the copy numbers were very different, CE predicted the two cycles accurately (Supplementary Figure S4B). Heterogeneity of ecDNA is not well studied and CE provides a method for its exploration in real data.

#### CE reconstructs heavier cycles in cancer cell-lines.

To test performance on cell-lines where ecDNA have been previously reported, we used utilized whole genome ONT reads and used CoRAL to generate an amplicon graph for 12 ecDNA amplicons. We compared the cycle extractions of CE, CE with connectivity-enforcement (CEc), and CoRAL on the amplicon graphs. Because the cycle reconstructions may differ and produce different orderings, we chose the sum of the length weighted copy number ratio (LWCNR) for the three heaviest cycles identified by each method. The sum LWCNR describes how much of the copy number of the amplified region is explained by the three heaviest cycles.

**Figure 3:**
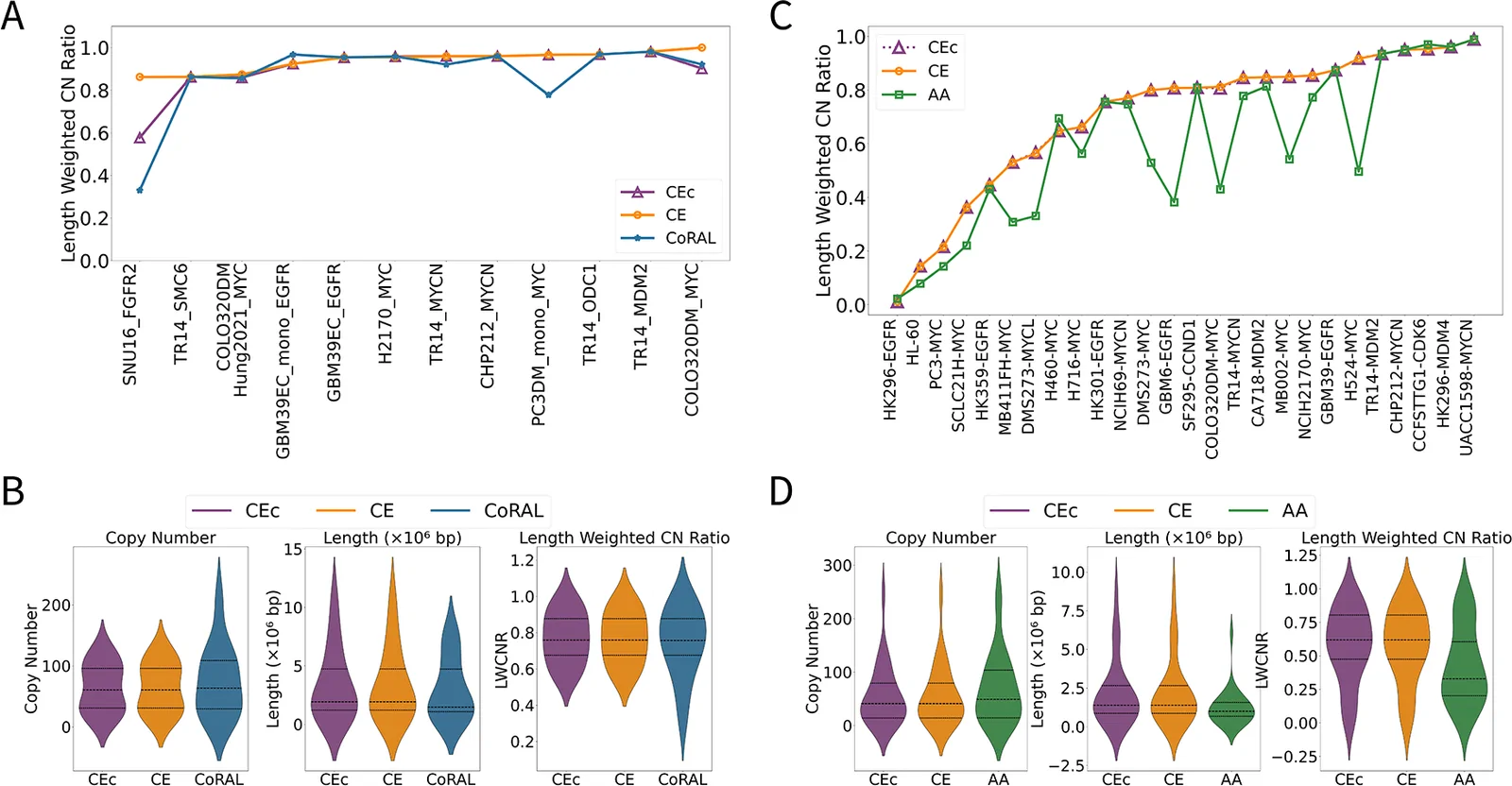
CE performance on cancer cell-lines. A. Comparison of LWCNR of the three heaviest cycles extracted by CE, CEc and CoRAL on ONT sequenced cell-lines. B. Copy Number, length and LWCNR of the heaviest cycle on ONT derived graphs. C. LWCNR of the 3 heaviest cycles extracted by CE, CEc and AA on Illumina sequenced cell-lines. D. Copy Number, length and LWCNR of the single heaviest cycle on Illumina derived graphs.

CE, CEc and CoRAL exhibited nearly identical behavior (Figure 3A), both selecting cycles with high LWCNR as their top-ranked cycles, and generally explaining a large fraction of the amplicon. SNU16 was the single exception where both CEc and CoRAL extracted only one cycle when the execution was stopped, while CE extracted 5 cycles and thus explained a larger proportion of LWCN. Notably, the one (heaviest) cycle extracted by CEc was identical to the heaviest cycle extracted by CE. In some other samples, CoRAL obtained lower LWCNR because it prioritized a heavy walk rather than a cyclic structure as the dominant amplicon. When considering all metrics, the total copy number, length, and LWCNR, comparing the three heaviest cycles is not meaningful, and we tested those for the single heaviest cycle. Here also, CE, CEc and CoRAL cycles matched up well (Figure 3B).

In contrast, when taking a short read amplicon graph as input, AA generally produced cycles with lower LWCNR (mean 0*.*598) compared to CE (mean 0*.*704) (Figure 3C,D). The performance of CE and CEc was effectively identical across all samples.

We also compared the performance of all methods using cell-lines for which both ONT and Illumina sequencing was available. Note that the copy number estimations, while comparable, are not identical between short and long-reads in these biological replicates, and LWCNR is not comparable. Therefore, we manually looked at LWCN, length, and CN of cycles produced on individual cell-lines (Supp. Figures S5-S9). In some cases (e.g., the CHP-212 ecDNA amplifying *MYCN*), the graph has a relatively simple structure. For simple cases, the cycles extracted by CoRAL and CE using the ONT graph and by AA and CE using the Illumina graph were all identical. In other cases, the cycles extracted by CoRAL and CE using the ONT amplicon graph were heavier and longer than the cycles extracted by AA and CE using Illumina. Moreover, even on the Illumina amplicon graph, the CE extracted cycle was heavier and longer than the AA extracted cycle.

As an additional test, we also evaluated tools that were designed for detecting smaller circular eccDNA, which are often < 10Kbp, and ubiquitously found in normal and cancer cells^17^. Specifically, we tested CReSIL^18^, which showed superior performance compared to other eccDNA tools^17^. In a small test on 2 simulated samples (where ground truth ecDNA structure was known), CReSIL did not find any ecDNA. On the cell-line CHP212, where a single ecDNA is known^27,44^, CReSIL missed the ecDNA but identified 115 smaller eccDNA, non-overlapping with the single ecDNA. Together, these results indicate the power of using long-reads and a principled cycle extraction algorithm for exDNA reconstruction.

#### CE runs 40× faster than CoRAL.

**Figure 4:**
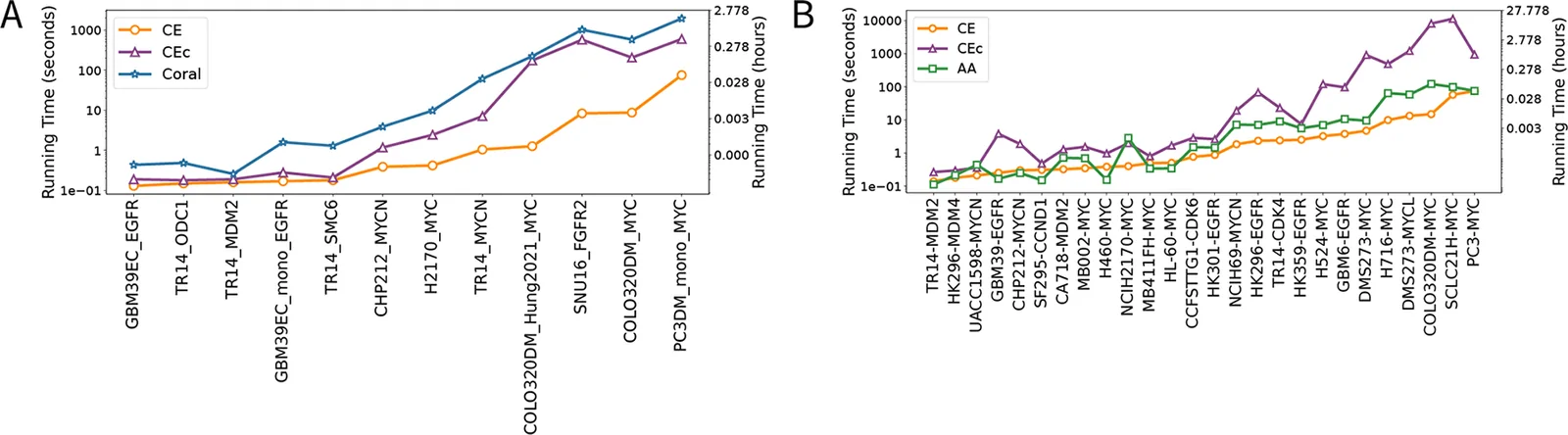
Running times of different methods for cycle extraction. A. Cycle extraction on ONT samples. B. Cycle extraction on Illumina samples. All cases, with the exception of SNU16 on ONT reads, terminated without reaching the time limit of 2 hours per iteration. The CoRAL and CEc running time for SNU16 are for extracting a single heaviest cycle.

CE was dramatically faster than other methods, completing in 0*.*13 to 75*.*47 seconds, with 58% of the samples finishing within one second (Figure 4A). In contrast, CoRAL required between 0*.*26 seconds to 32*.*5 minutes, and its mean running-time was 40× higher than CE. The running time of CEc was between 0*.*18 second to 10 minutes. CEc finished running 42% of samples within 1 second. Notably, all cases, with one exception, terminated without reaching the time limit of 2 hours per iteration. SNU16 was one challenging example with 71 discordant edges and 338 edges in total, where CoRAL and CEc extracted a single heaviest cycle before termination, and we reported the time for extracting the first cycle. However, CE only took 8.37 seconds to extract 5 cycles which explained 90.73% LWCN of the graph.

The results on Illumina short-reads were similar. CE continued to be the fastest method, running faster than AA on 75% of the samples despite the MILP based cycle extraction procedure (Figure 4B), while maintaining much higher reconstruction quality (heaviness). For CEc, application of connectivity enforcement method introduces additional (though, increasing linearly in the graph size) integer variables, increasing CEc running time compared to CE.

The running time of CEc is higher when using the short-read graph compared to its time on the long-read graph. Because of missing breakpoints, the cycles extracted are shorter, and more cycles are extracted, leading to higher running time for CEc using the short-read graph compared to the long-read graph.

On simulated data, the average running time after graph construction was 0.31 seconds for CE, 3.69 seconds for CoRAL, and 53.6 seconds for Decoil. Notably, both CE and CoRAL support multithreading, whereas Decoil does not. To ensure a fair comparison, the average running times calculate for CE and CoRAL were therefore measured using a single thread. On both simulated long-read data and cell lines, the running time of CE increased with graph complexity (i.e., the number of edges, Supplementary Figure S10A,B), though it was not the sole determinant. For example, CE resolved the PC3 MYC amplicon with 310 edges (133 sequence, 118 concordant, 59 discordant) in 75 seconds, while the larger SNU16 graph (338 edges in total, 138 sequence, 129 concordant, 71 discordant) was solved in under 10 seconds. This is because CE identified multiple disconnected cycles early in the SNU16 iterations, reaching the termination criterion (90% graph LWCN coverage) more quickly.

#### CE reconstructions improve our understanding of ecDNA biology.

The algorithmic approach utilized by CE improves upon our ability to reconstruct ecDNA sequences, both on short reads, and also on long-reads where additional subwalk constraints can be satisfied. The tested cell lines included GBM39, TR14, H2170, which have been previously studied, and our results matched the previously predicted structures exactly^15,26,28^.

**Figure 5:**
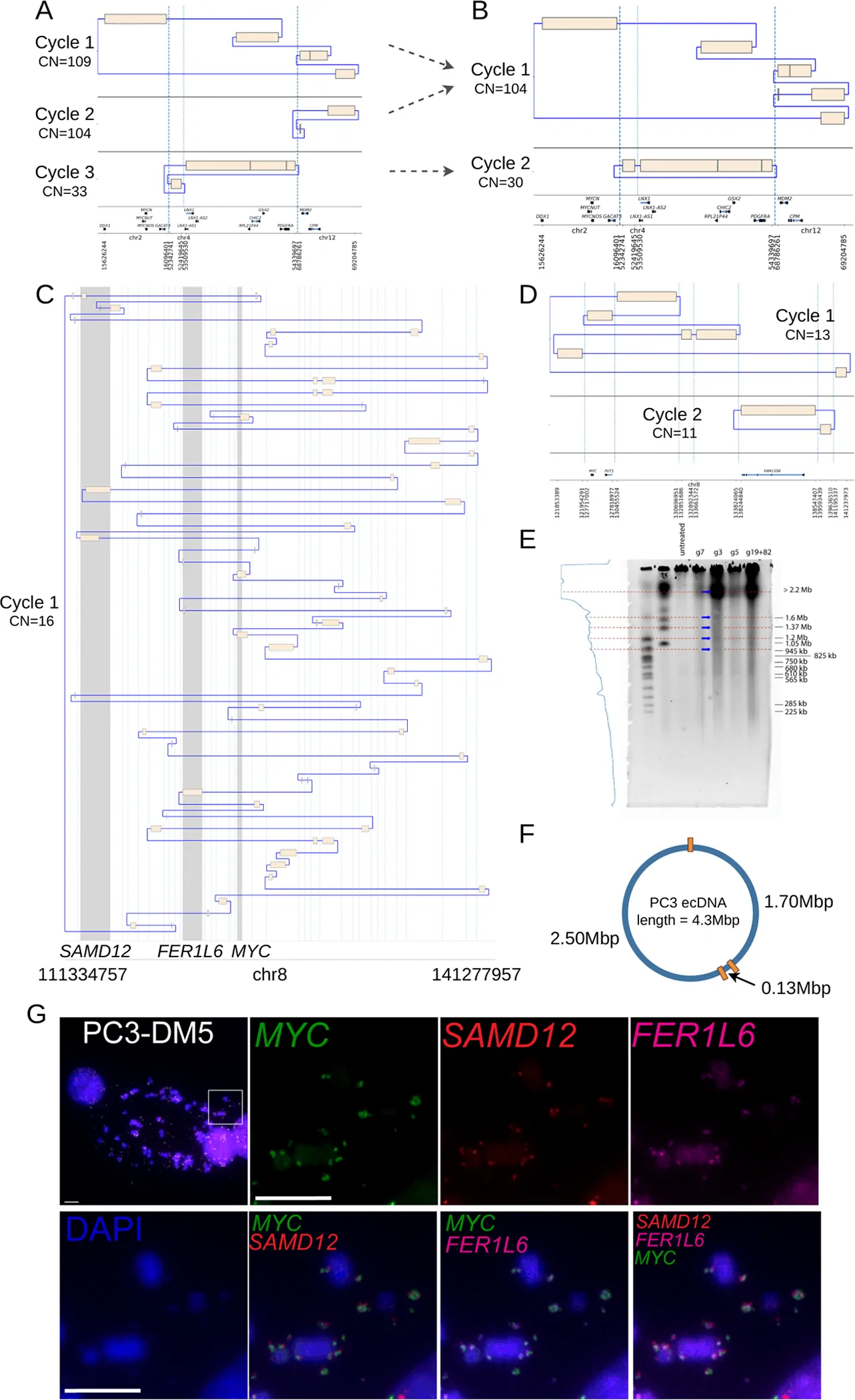
AA and CE reconstructions on specific cell-lines. A. AA reconstruction with 3 cycles for CA718. B. CE reconstruction of CA718 ecDNAs outputs 2 cycles. C. CE reconstruction for PC3 amplicon. D. CE reconstruction of PC3 using the AA (Illumina) graph. E. CRISPR Catch data for CE amplicon suggests fragments of lengths > 2*.*2Mbp and 1*.*6Mbp. F. Schematic reconstruction of the PC3 ecDNA with gRNA sites marked. G. DNA FISH validation using probes for *Myc, SAMD12*, and *FER1L6* loci. Co-ocurrence of the 3 probes supports the CE long-read reconstruction.

We next investigated cases where the CE predictions were different from previous predictions. For Illumina reads acquired from the line CA718, AA predicted 3 cycles, one carrying *MYCN*and *MDM2*, another carrying *PDGFRA*, and a third, non-genic cycle (Figure 5B). In contrast, CE identified 2 cycles, effectively merging the non-genic cycle of AA with the *MDM2*+*MYCN* cycle (Figure 5B) to generate a longer and heavier cycle. The CE reconstruction is meaningful and suggests that predictions of non-genic ecDNA containing only regulatory sequences^4^ should be orthogonally validated. We also observed that the two cycles predicted by CE (and by AA) share genomic segments. Our reconstructions do not preclude the presence of alternative species obtained by recombination across the shared fragments.

We also compared the CE cycles generated on a PC3 amplicon using Nanopore and Illumina sequencing. The CE cycle reconstructed from Nanopore was substantially larger and more amplified than those obtained from the short-read data, predicting a 4.32 Mbp cycle (Figure 5C) explaining 78% of the amplicon copy number. In contrast, the cycle extracted using Illumina reads (Figure 5D) had length 690 Kbp and explained 9% of the amplicon. The CN of the oncogene *MY C* was 12 copies in the short-read cycle versus 47 copies in the long-read cycle. To validate our data, we performed a CRISPR-Catch experiment^38^ where single CRISPR mediated cuts linearized the ecDNA, and pulse-field gel electrophoresis was used to separate the linearized product. Multiple guide RNAs were used to linearize the structure, and produced fragments with different lengths (Figure 5E). Notably, gRNA g3 produced fragments of lengths > 2*.*2, and 1*.*7 Mbp. On the CE reconstruction of PC3 ecDNA, g3 occurred thrice, and supported fragments of size 2*.*5, 1*.*7, and 0*.*13 Mbp (Figure 5F), consistent with the CRISPR-Catch data. The gRNA g5 is contained in the oncogene *MYC*. It appeared twice on the cycle and supported fragments of size 0*.*12 and 4*.*3 Mbp. The other gRNA were controls which did not appear on the predicted cycle. All of these were consistent with the CRISPR-Catch data.

The CE-reconstructed ecDNA using long-reads included many loci that were not in the short-read reconstruction, for example in regions encoding *SAMD1* and *FER1L6*, while also containing the *MYC* locus that was originally reported for short-read reconstruction (Figure 5C; shaded regions). To validate, we performed a DNA multi-FISH experiment (Supplementary Methods), with probes for the 3 loci. The results showed co-occurrence of the probes in multiple ecDNA (Figure 5C), supporting the reconstruction.

## 4 Discussion

Reconstruction of the ecDNA sequence is important to understand the content of ecDNAs identified in cancer genomes. Nevertheless, it remains a very challenging problem for two reasons. First, ecDNA can carry multiple larger regions with higher multiplicity, which is akin to the problem of repeats in genome assembly, but harder because the regions of high multiplicity are very large often exceed 100 Kbp.

We describe an algorithmic approach wherein a cycle is extracted from the amplicon graph that best explains the copy number and breakpoints represents the current structure. Unlike previous methods, the CE methods utilizes an MILP optimization that is an order of magnitude faster while maintaining accuracy. Moreover, CE is designed to work off both Illumina and ONT reads and can be easily adapted to other sequencing technology. On long-reads, it additionally optimizes the use of subwalk-constraints for better disambiguation of sequences. For both short and long read, the input amplicon graphs required as input to CE are now readily produced from wgs data using robust available tools^31,35^, making it seamless to use.

In extensive tests, CE reconstructed ecDNA structures with high accuracy. Some metrics such as LWCNR could favor CE, which optimizes length weighted copy number (LWCN). However, when the ground truth (the true cycle) was known, for example in simulated data, we only used metrics that were independent of CE’s objective function, thus providing a fair comparison for all methods.

When ground truth was not known, for example in cell-lines, LWCNR was a reasonable evaluation metric. It did not require access to ground truth data, and higher values suggest that a larger fraction of the amplified region was explained by the predicted ecDNA, providing a parsimonious explanation. For many well studied cell lines, we obtained the identical answer to the previous (often manual) reconstructions from earlier studies. In one case (PC3-DM), where we obtained a substantially novel reconstruction, we performed CRISPR Catch and DNA FISH experiment for orthogonal validation. The results strongly supported the CE reconstructions, and provided support for the CE objective function.

In ongoing future work, we will create modules for AmpliconSuite and CoRAL so that cycle extraction can be automatically performed using CE. By determining the ecDNA sequence, CE will have direct utility for investigating functional aspects of ecDNA including enhancer hijacking^27^, regulatory rewiring, presence and absence of genes^4,5^ and other functional elements, the DNA repair mechanisms used^45^, evolution of ecDNA structure^6^ and others.

The second challenge with ecDNA reconstruction is the heterogeneity where multiple ecDNA share genomic segments, and it is not clear if they represent the same ecDNA or different ones. Notably, those ecDNA could exist and function similarly as a single molecule or multiple molecules, or even both in one sample. Thus, even when heterogeneity exists, it is not easy to formulate as a problem of cycle extraction. In preliminary results, CE can extract two ecDNA sequences with shared segments if they have distinct copy numbers. An example of this is in the CA718 reconstruction in Figure 5B, where the CE reconstruction does not preclude the existence of other, less abundant species formed by recombination across the shared genomic segment. We plan to extend our methods by developing the problem not as one of reconstruction but rather about estimating the level of heterogeneity in terms of conflicting subwalk constraints.

In general, unambiguous determination of the correct ecDNA sequence will be best achieved by a combination of sequencing and other experimental methods. These include single molecule sequencing extensions such as CRISPR-Catch^38^, single-cell methods like ATAC-seq^46^ and Hi-C^28,47^ and orthogonal technologies such as DNA-FISH^31^ and others. These methods can be applied to individual samples of interest, and future work will seek to combine these methods with the algorithmic analysis of amplicon graphs. In the meantime, CE can serve as an easy to use and relatively accurate method for ecDNA reconstruction.

## Supplementary Material

Supplement 1

## Footnotes

1 While CE prioritizes cyclic walks to reconstruct ecDNA, it can also identify non-cyclic walks when no heavy cycles can be found (see Supplementary Methods S1.3 for details).

## 5 Code Availability

The Cycle Extractor source code is publicly available under an open-source license at: https://github.com/AmpliconSuite/CycleExtractor. The repository includes detailed documentation, example datasets, and scripts for reproducing key results presented in this study.
