# Supplementary material for: Fast and accurate resolution of ecDNA sequence using Cycle-Extractor
Source: bioRxiv. 2026 Apr 15:2026.03.10.710955. Preprint. [Version 2] doi: 10.64898/2026.03.10.710955 (PMC13105081; doi:10.64898/2026.03.10.710955)
Supplement: Supplementary file 1 [file NIHPP2026.03.10.710955v2-supplement-1.pdf]

bioRxiv preprint doi: https://doi.org/10.64898/2026.03.10.710955; this version posted April 15, 2026. The copyright holder for this preprint (which was not certified by peer review) is the author/funder, who has granted bioRxiv a license to display the preprint in perpetuity. It is made available under a CC-BY 4.0 International license.

# Supplementary Figures

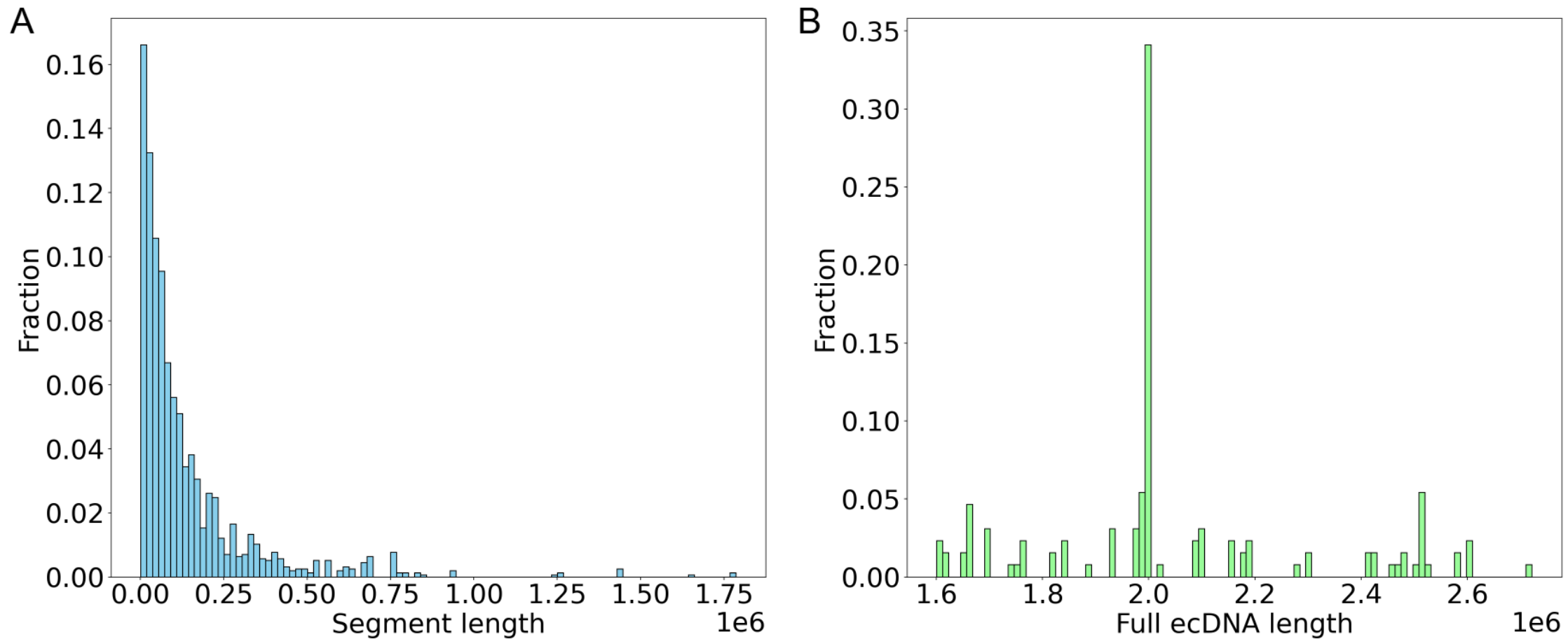


Supplemental Figure S1: **The lengths of the segments versus the lengths of the ecDNA.** The large majority of the segments are smaller than 250K, while ecDNA are larger, with average size exceeding 2Mbp.

bioRxiv preprint doi: https://doi.org/10.64898/2026.03.10.710955; this version posted April 15, 2026. The copyright holder for this preprint (which was not certified by peer review) is the author/funder, who has granted bioRxiv a license to display the preprint in perpetuity. It is made available under a CC-BY 4.0 International license.

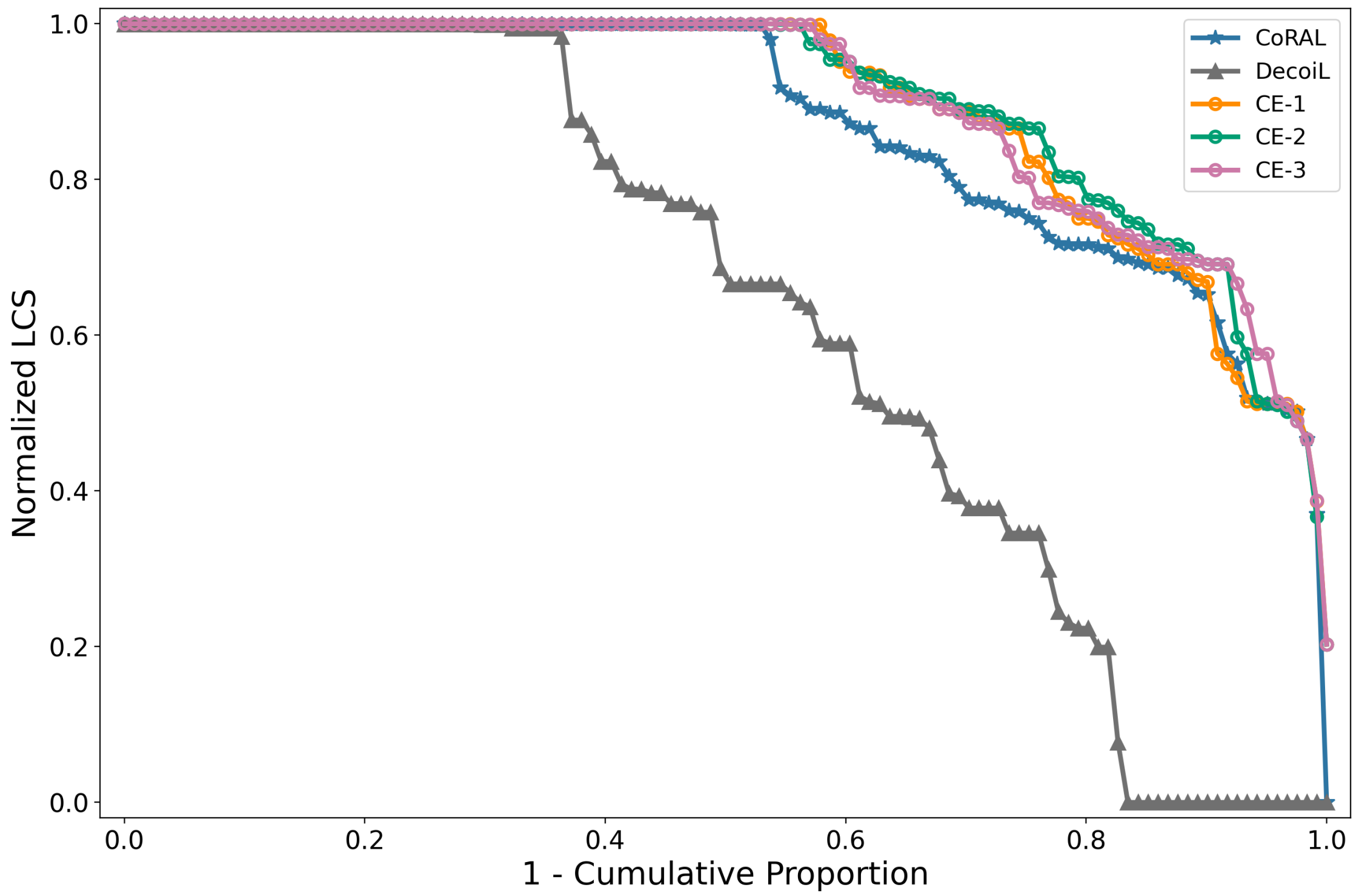


Supplemental Figure S2: **Stability of cycle ordering under randomized traversal** Cumulative distribution of normalized longest common subsequence (LCS) values obtained from three runs of the traversal step with different random orders on the same optimized edge set. Curves CE-1, CE-2, and CE-3 correspond to three independent runs. The LCS metric for CoRAL and Decoil are added for comparison here.

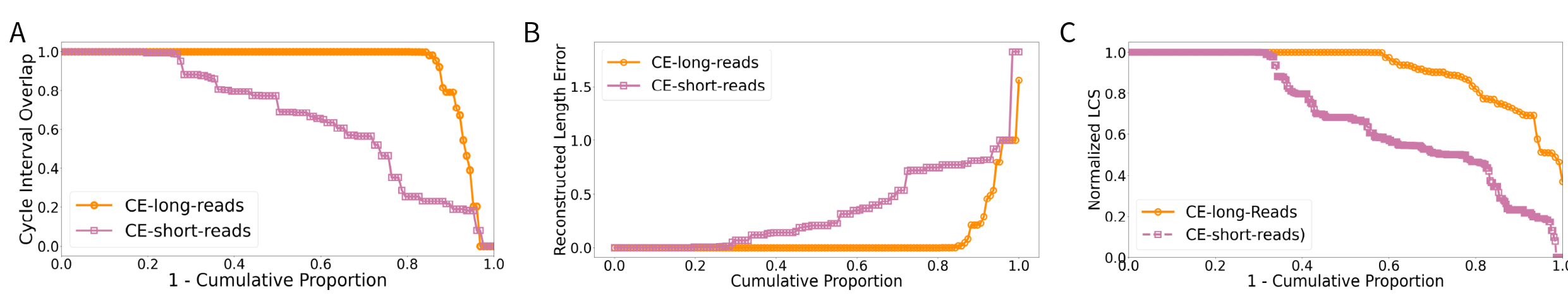


Supplemental Figure S3: **Comparison of A. CIO, B. RLE and C. LCS metrics by CE on simulated data using long-read and short-read graphs.**

bioRxiv preprint doi: https://doi.org/10.64898/2026.03.10.710955; this version posted April 15, 2026. The copyright holder for this preprint (which was not certified by peer review) is the author/funder, who has granted bioRxiv a license to display the preprint in perpetuity. It is made available under a CC-BY 4.0 International license.

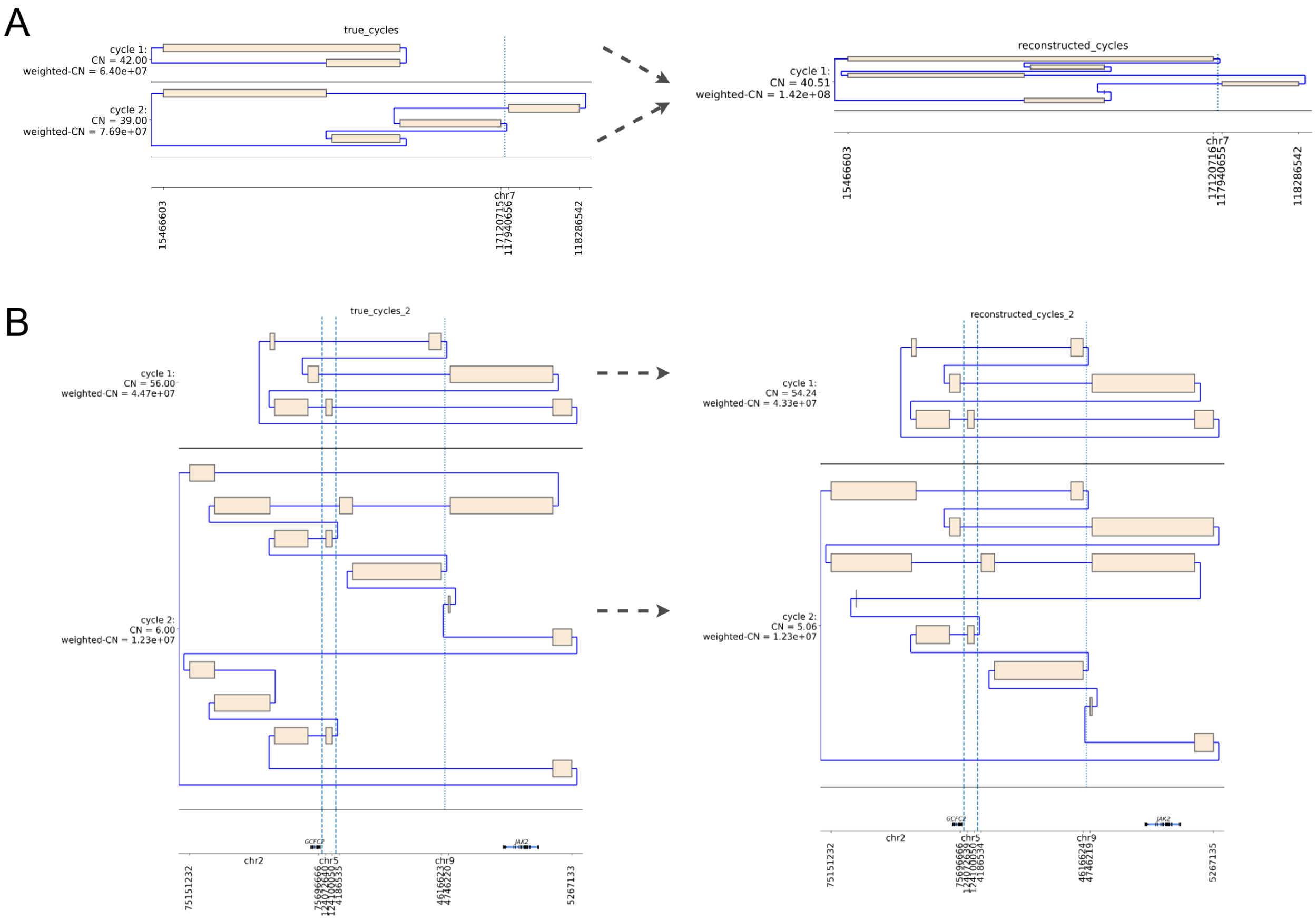


Supplemental Figure S4: **CE behavior on heterogeneous data.** A. CE merges two true cycles onto one when they share genomic segments and are very similar in copy number. B. CE predicts two separate cycles when the true cycles have very different copy numbers.

bioRxiv preprint doi: https://doi.org/10.64898/2026.03.10.710955; this version posted April 15, 2026. The copyright holder for this preprint (which was not certified by peer review) is the author/funder, who has granted bioRxiv a license to display the preprint in perpetuity. It is made available under a CC-BY 4.0 International license.

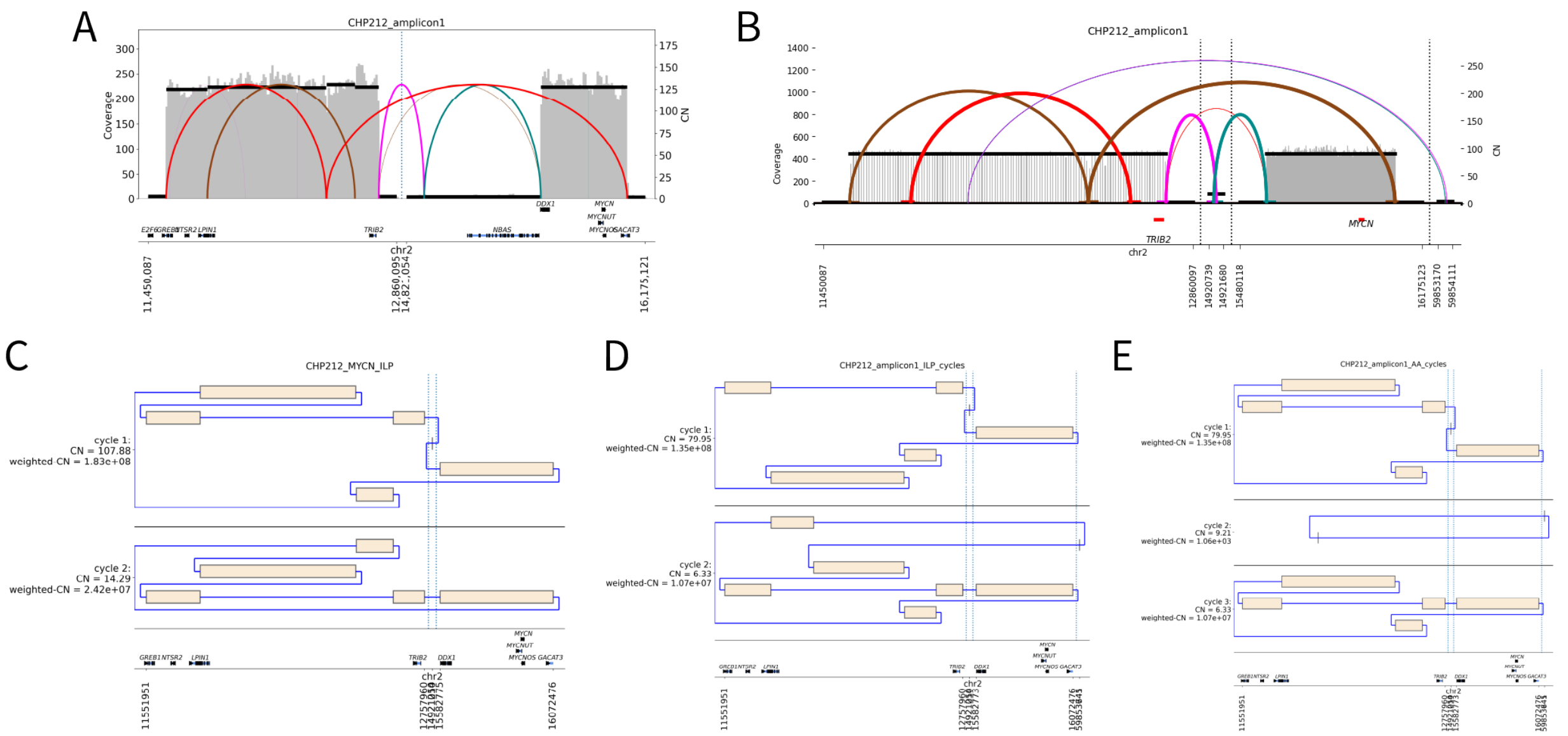


Supplemental Figure S5: **CE reconstruction on CHP-212 cell line.** A. Long read amplicon graph. B. Short read amplicon graph. C. CE reconstruction using long-read graph with 2 cycles, which is identical to CoRAL. D. CE reconstruction using short-read graph. E. AA reconstruction (AA can only extract cycles from short-read graph) with 3 cycles.

bioRxiv preprint doi: https://doi.org/10.64898/2026.03.10.710955; this version posted April 15, 2026. The copyright holder for this preprint (which was not certified by peer review) is the author/funder, who has granted bioRxiv a license to display the preprint in perpetuity. It is made available under a CC-BY 4.0 International license.

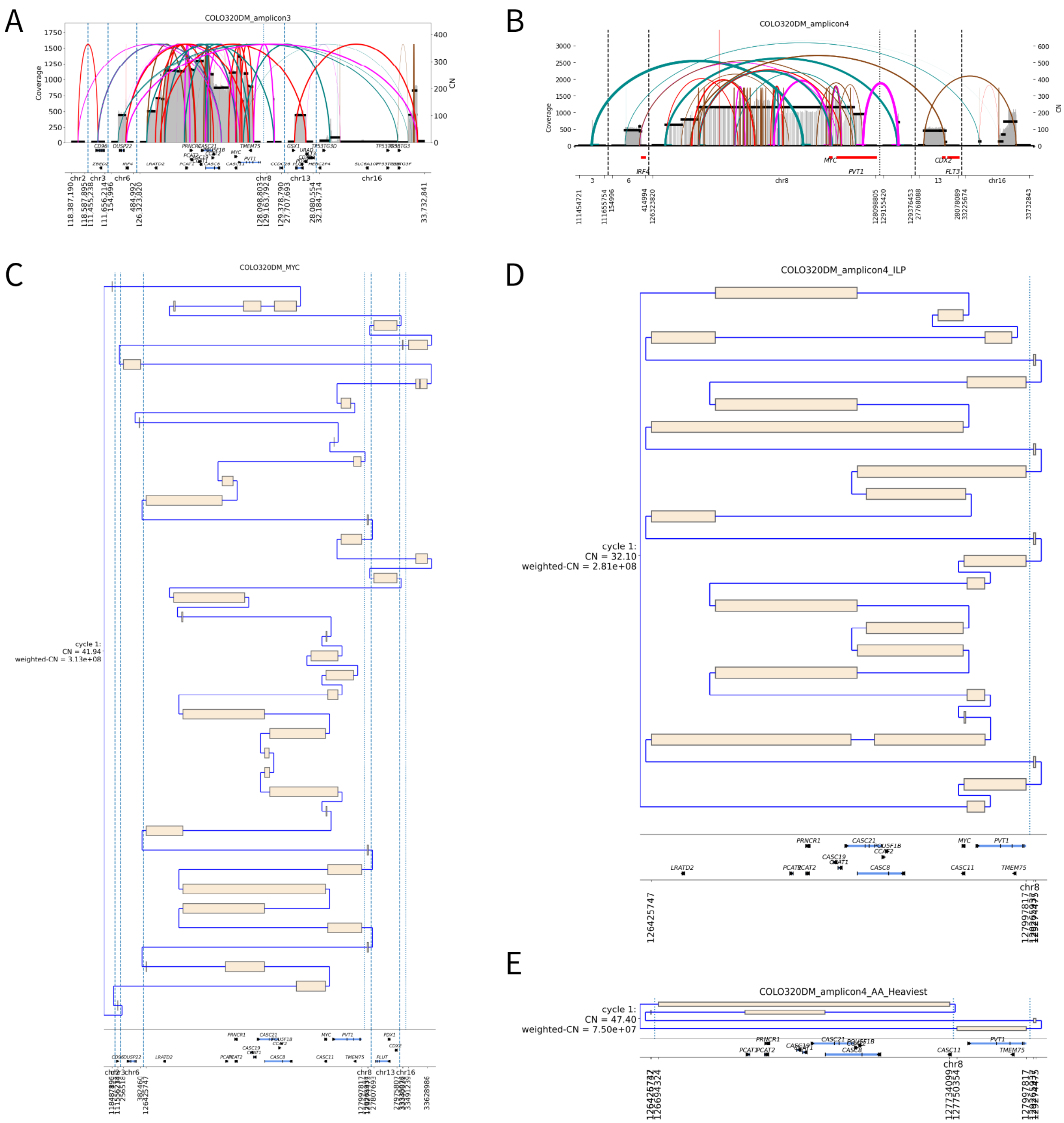


Supplemental Figure S6: **CE reconstruction on COLO320DM cell line.** A. Long read amplicon graph. B. Short read amplicon graph. C. The heaviest cycle from CE reconstruction using the long-read graph, which is identical to CoRAL. D. The heaviest cycle from CE reconstruction using short-read graph. E. The heaviest cycle from AA reconstruction.

bioRxiv preprint doi: https://doi.org/10.64898/2026.03.10.710955; this version posted April 15, 2026. The copyright holder for this preprint (which was not certified by peer review) is the author/funder, who has granted bioRxiv a license to display the preprint in perpetuity. It is made available under a CC-BY 4.0 International license.

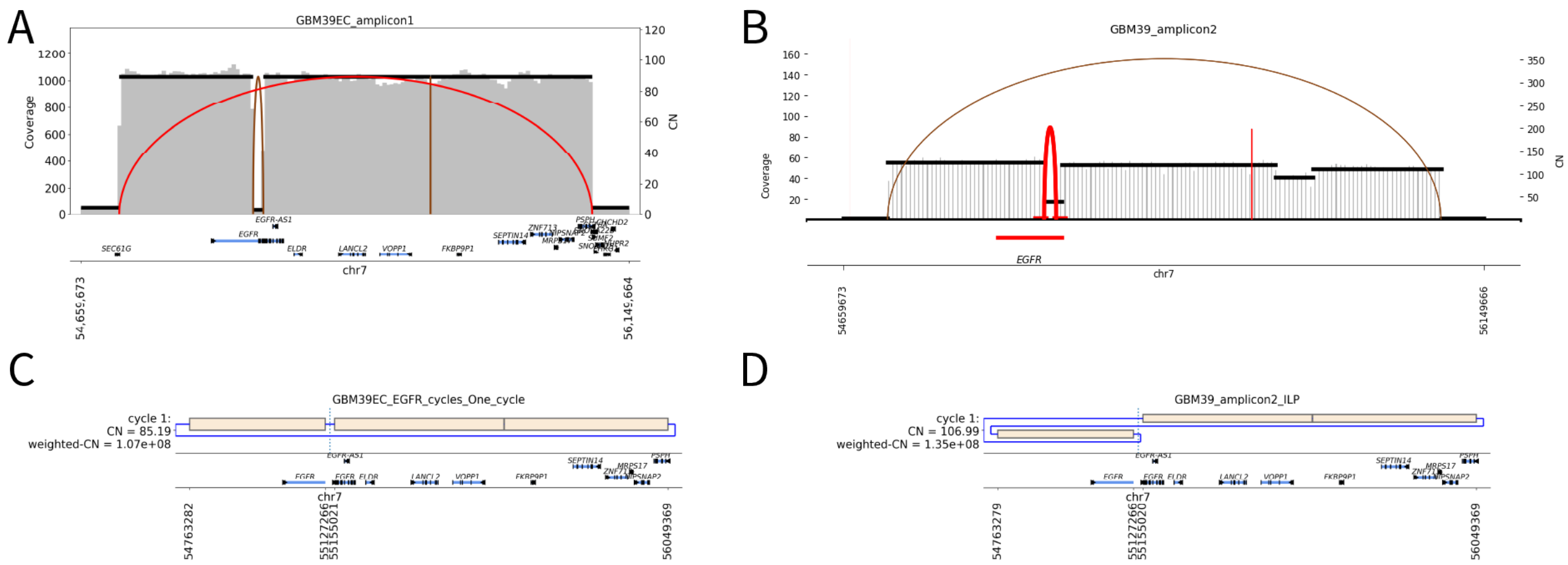


Supplemental Figure S7: **CE reconstruction on GBM39 cell line.** A. Long read amplicon graph. B. Short read amplicon graph. C. The heaviest cycle from CE reconstruction using the long-read graph, which is identical to CoRAL. D. The heaviest cycle from CE reconstruction using the long-read graph, which is identical to AA.

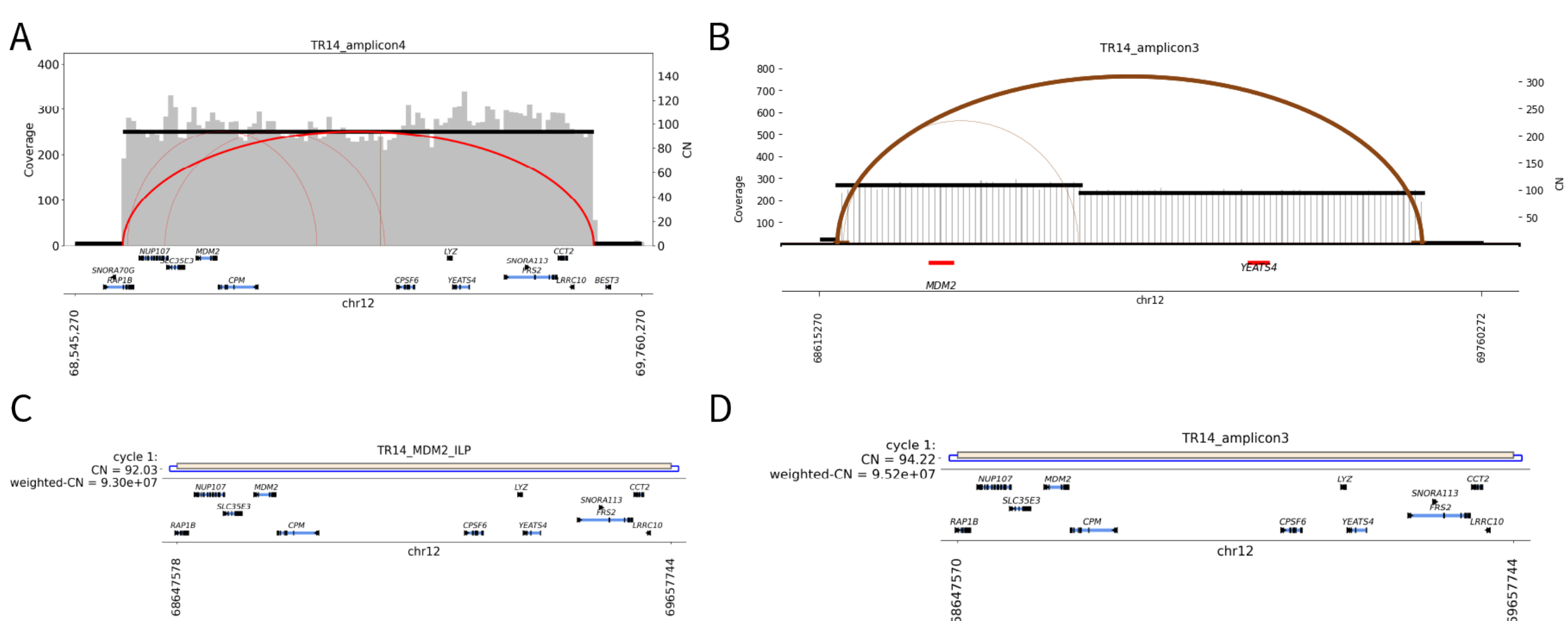


Supplemental Figure S8: **CE reconstruction on TR14 cell line, *MDM2* amplicon.** A. Long read amplicon graph. B. Short read amplicon graph. C. The heaviest cycle from CE reconstruction using the long-read graph, which is identical to CoRAL. D. The heaviest cycle from CE reconstruction using the long-read graph, which is identical to AA.

bioRxiv preprint doi: https://doi.org/10.64898/2026.03.10.710955; this version posted April 15, 2026. The copyright holder for this preprint (which was not certified by peer review) is the author/funder, who has granted bioRxiv a license to display the preprint in perpetuity. It is made available under a CC-BY 4.0 International license.

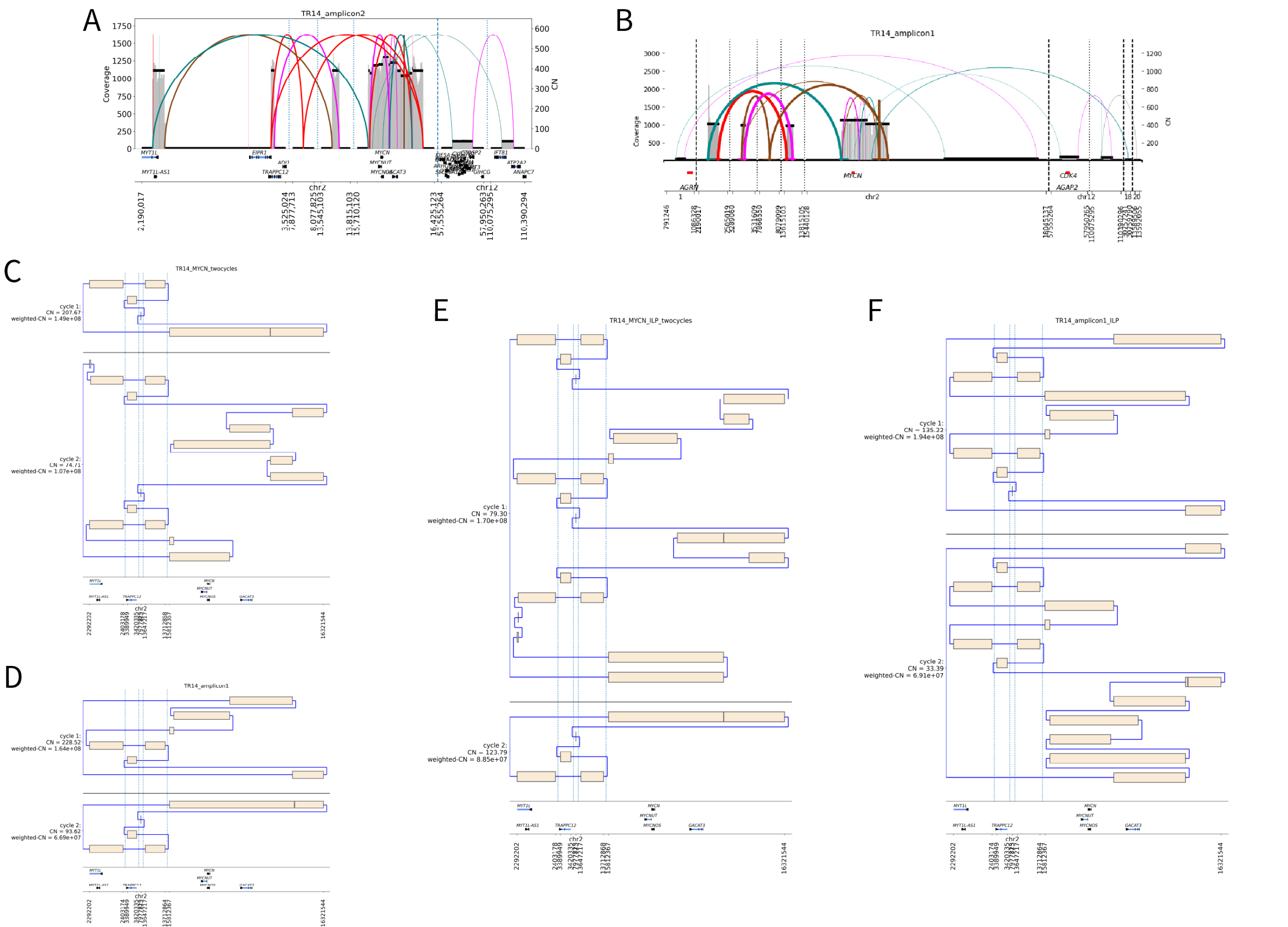


Supplemental Figure S9: **CE reconstruction on TR14 cell line, *MYCN* amplicon.** A. Long read amplicon graph. B. Short read amplicon graph. C. 2 heaviest cycles from CoRAL reconstruction. D. 2 heaviest cycles from AA reconstruction. E. 2 heaviest cycles from CE reconstruction, using long read graph. F. 2 heaviest cycles from CE reconstruction, using short read graph.

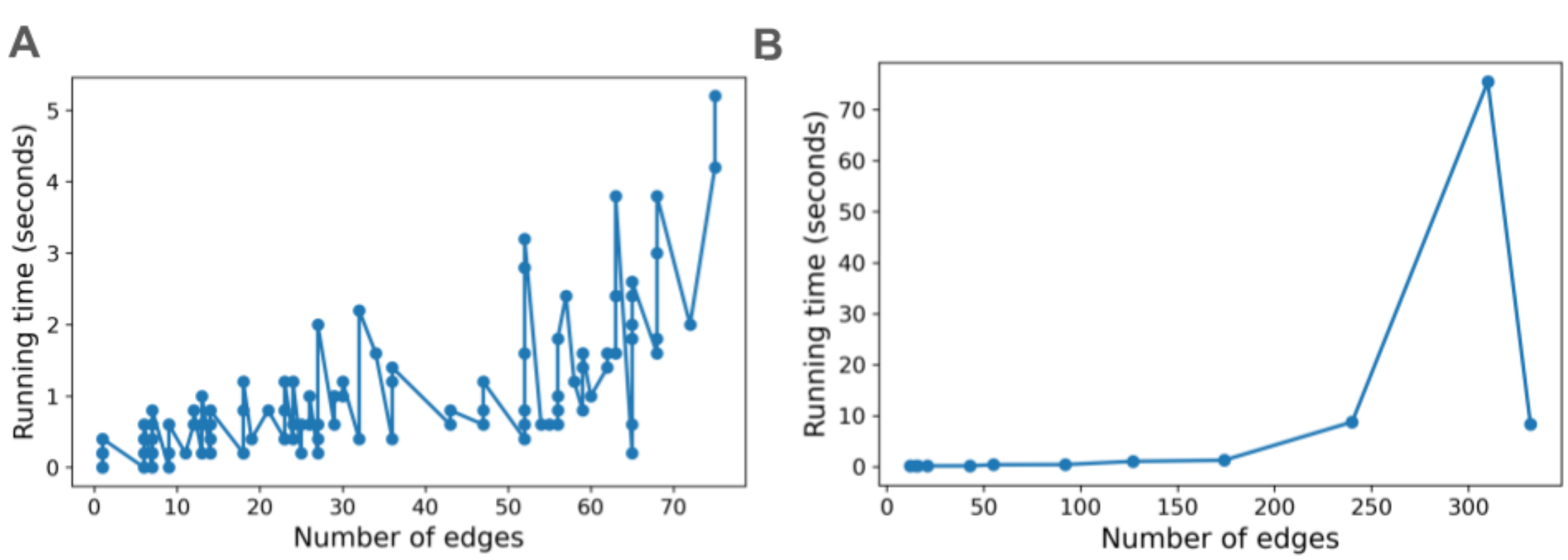


Supplemental Figure S10: **Running time vs the number of edges of the graph.** A. CE running time vs number of edges for simulated long-read data from cell lines. B. CE running time for long-read (ONT) data on cell lines.

bioRxiv preprint doi: https://doi.org/10.64898/2026.03.10.710955; this version posted April 15, 2026. The copyright holder for this preprint (which was not certified by peer review) is the author/funder, who has granted bioRxiv a license to display the preprint in perpetuity. It is made available under a CC-BY 4.0 International license.

# S1 Supplementary Methods

## S1.1 Full ILP for the *optimization* step

The full CE MILP formulation is provided below.

**Parameters.**

$M$**:** maximum multiplicity allowed for concordant and discordant edges

$\gamma(W)$**:** $= 0.01 \sum_{(u,v)\in W} C_{uv}\ell_{uv}$, and balances weights of subwalk constraints relative to LWCN in the objective.

$p^j_{uv} \in \{0,1\}$ : indicates if edge $(u,v)$ is in subwalk $p_j$

**Variables.**

$F \in \mathbb{R}$ : copy number assigned to edges in the solution but with multiplicity one.

$x_{uv} \in \{0,1\}$ : $x_{uv} = 1$ if edge $(u,v)$ is selected, and 0 otherwise.

$f_{uv} \in \mathbb{R}$ : copy number assigned to edge $(u,v)$ in the solution, should be multiples of $F$.

$y_{uvm} \in \{0,1\}$ : (auxiliary) $y_{uvm} = 1$ iff $(u,v)$ has multiplicity $m \in \{1 \le m \le M\}$ for each discordant edge in a feasible walk.

$z_{uvm} \in \mathbb{R}$ : The auxiliary variable is used to constrain $z_{uvm} = m \cdot F \cdot y_{uvm}$ which is a quadratic formula but is linearized later.

$P_j \in \{0,1\}$ : indicates if subwalk $p_j$ is satisfied $1 \le j \le p$.

$d_u \in \{0,1,2,..\}$ : indicates the order of the nodes, used in connectivity constraints.

$c^+_{uv} \in \{0,1\}$ : be a binary variable that is 1 if the direction is from $u$ to $v$, again used in connectivity constraints.

**CE MILP.** CE maximizes the sum of LWCN and $\gamma$-weighted subwalk constraint using

$$\max \sum_{(u,v)\in E} f_{uv}\ell_{uv} + \gamma \frac{\sum_j P_j}{|\mathcal{P}|}$$

while satisfying the following constraints.

1. **capacity**: For any edge $(u,v)$ in a feasible solution, its copy number $(f_{uv})$ must not exceed its capacity $(C_{uv})$. If the edge is not chosen in the solution, its copy number is zero.

$$f_{uv} \le C_{uv} \cdot x_{uv} \ \ \forall (u,v) \in E$$

2. **balance**: The sum of the solution copy number of discordant and concordant edges that connect to a node is equal to the solution copy number of the sequence edge connected to that node.

$$\sum_{(w,u)\in E_c \cup E_c} f_{wu} = \sum_{(u,v)\in E_s} f_{uv}$$

3. **copy number $F$ with multiplicity one**: Note that if edge $(u,v)$ is picked in the solution (i.e., $x_{uv} = 1$), then $F \le f_{uv}$, but otherwise $(u,v)$ imposes no constraint. For this purpose we introduce a sufficiently large constant $C_{\max}$ (e.g., $C_{\max} = M \cdot \max_{(u,v)\in E} f_{uv}$).

$$F \le f_{uv} + C_{\max} \cdot (1 - x_{uv}) \ \ \forall (u,v) \in E_c \cup E_d$$

bioRxiv preprint doi: https://doi.org/10.64898/2026.03.10.710955; this version posted April 15, 2026. The copyright holder for this preprint (which was not certified by peer review) is the author/funder, who has granted bioRxiv a license to display the preprint in perpetuity. It is made available under a CC-BY 4.0 International license.

4. **discordant edge multiplicity constraint**: The multiplicity of a discordant edge is managed using

$$\sum_{m=1}^{M} y_{uvm} = x_{uv} \qquad \forall (u,v) \in E_d.$$

Subsequently the copy of number of the edge is given by the (quadratic) constraint

$$f_{uv} = \sum_{m=1}^{M} m \cdot F \cdot y_{uvm} = \sum_{m=1}^{M} z_{uvm} \qquad \forall (u,v) \in E_d$$

which guarantees $f_{uv}$ are multiples of $F$. We linearize the quadratic constraints using

$$\begin{aligned} z_{uvm} \leq&\ mF \\ z_{uvm} \leq&\ C_{\max} \cdot y_{uvm} \\ z_{uvm} \geq&\ mF - C_{\max} \cdot (1 - y_{uvm}) \\ z_{uvm} \geq&\ 0 \end{aligned}$$

5. **subwalk constraints**: If a subwalk is in the solution ($P_j = 1$), all of its edges are in the solution. If a subwalk in not in the solution ($P_j \neq 0$), there is not constraint here.

$$x_{uv} \geq P_j \cdot p^j_{uv} \qquad \forall j \in \{1, ..., p\} \qquad \forall (u,v) \in E \cap p_j$$

6. **connectivity constraints 1 (start node):** Assign $s$ to be the start node.
The order of $s$ is 1.

$$d_s = 1$$

No direction into $s$.

$$\sum_v c^+_{vs} = 0$$

Direction out of s

$$c^+_{sv} \leq \sum_v x_{sv}$$

7. **connectivity constraints 2 (order of nodes):** If node $u$ is not in the solution, then $d_u = 0$, otherwise $d_v$ is a positive integer which reflects the order of the node.

$$d_u \leq |V| \sum_v x_{uv} \qquad \forall u \in V$$

8. **connectivity constraints 3 (edge direction):** Only one direction from each edge in the solution

$$c^+_{uv} + c^+_{vu} \leq x_{uv} \qquad \forall u, v \in V$$

9. **connectivity constraints 4 (incoming edge for nodes):** If a node is part of the solution, and it is not $s$ (the starting node), then it must have at least one incoming direction

$$\sum_v c^+_{vu} \geq \frac{1}{|V|} \sum_v x_{uv} \qquad \forall u \in V - \{s\}$$

10. **connectivity constraints 5:**

$$d_v - d_u \geq 1 - |V|(1 - c^+_{uv}) \ \ \forall u, v \in V$$

bioRxiv preprint doi: https://doi.org/10.64898/2026.03.10.710955; this version posted April 15, 2026. The copyright holder for this preprint (which was not certified by peer review) is the author/funder, who has granted bioRxiv a license to display the preprint in perpetuity. It is made available under a CC-BY 4.0 International license.

## S1.2 Comparison of CE and CoRAL formulations

For completeness, we summarize and compare the formulations of CoRAL and CE (see Supplementary Table S1). In CoRAL (specifically the max weight formulation), the objective function was quadratic. CoRAL comprised 20 classes of constraints; of which 4 were quadratic and the others were linear. We reformulated the same optimization problem with 16 classes of MILP constraints. One key difference between CE and CoRAL is the treatment of edge multiplicities. In CE, we do not introduce an explicit multiplicity variable for edges. Instead, the model includes a variable representing the copy number of the ecDNA cycle, along with variables representing the copy number contributed by each edge. The multiplicity of an edge can then be calculated after solving the MILP optimization by dividing the edge copy number by the copy number of the ecDNA cycle. In contrast, CoRAL directly models edge multiplicities as integer variables within its optimization framework.

| Model | CE | CoRAL |
|---|---|---|
| Objective | **Linear** | Quadratic |
| Eulerian constraint: cycle/walk(s) can be traversed by alternating sequence and breakpoint edges | Linear | Linear |
| CN Constraint: total CN of edge $e$ in the cycle/walk $\leq$ remaining CN($e$) $\forall$ edge $e$ | **Linear** | Quadratic |
| Multiplicity constraint: repeat times of edge $e \leq R(e)$ for each discordant edge $e$ | Linear | Linear |
| Subwalk constraints | Linear | Linear |
| Connectivity constraint | **Linear (optional)** | Quadratic |

Supplementary Table S1: Comparison of objective and constraint types (linear vs quadratic) in CE and CoRAL.

## S1.3 Integer multiples of $F$ of all edges in the solution

**Lemma S1.1.** *If the copy numbers of the discordant edges in the cycle are positive integer multiples of $F$ (as enforced by Constraint 4), then the copy numbers of the concordant and sequence edges in the solution cycle are also positive integer multiples of $F$.*

*Proof.* We consider a canonical ordering of all nodes in the MILP solution based on their chromosomes and then on their nucleotide coordinates. By definition, node 1 in the solution is connected to a sequence edge and one or more discordant edges, but not to any concordant edge. We prove by induction that for each node $k$ in the ordering,any edge incident on node $k$ must have a copy number that is an integer multiple of $F$.

**Base case ($k = 1$).** We know that node 1 is only incident on a single sequence edge, and all other edges connecting to 1 are discordant, with copy numbers that are multiples of $F$. By the balance condition, the sequence edge $(1, 2)$ must also have a copy number that is an integer multiple of $F$. Therefore, all edges connected to node 1 have copy numbers that are positive integer multiples of $F$.

**Induction hypothesis.** Assume that every edge incident to nodes $1, 2, ..., k$ has copy number equal to a positive integer multiple of $F$.

**Inductive Step**. We now consider node $k + 1$. All discordant edges incident on node $k + 1$ must have copy numbers that are multiples of $F$, and we need to investigate the concordant and sequence

bioRxiv preprint doi: https://doi.org/10.64898/2026.03.10.710955; this version posted April 15, 2026. The copyright holder for this preprint (which was not certified by peer review) is the author/funder, who has granted bioRxiv a license to display the preprint in perpetuity. It is made available under a CC-BY 4.0 International license.

edges (denoted $e_c, e_s$, respectively) incident on $k+1$. We have two cases.

First, suppose $\text{CN}[e_c] = 0$. Then, by the balance condition, $\text{CN}[e_s]$ must be an integer multiple of $F$. Second, we consider the case $\text{CN}[e_c] > 0$, then both nodes must be a part of the solution, and by our ordering criteria, $e_c = (k, k+1)$, connects node $k$ to $k+1$. Then, using induction hypothesis, $\text{CN}[e_c]$ must be an integer multiple of $F$, and by balance, $\text{CN}[e_s]$ must be a positive integer multiple of $F$.

□

## S1.4 Implementation details

**Preprocessing amplicon graphs.** While CE accepts an amplicon graph directly from the output of AA[29] or CoRAL[35], it replaces each foldback edge $(u, u) \in E_d$ with $(u, u_1) \in E_d$, $(u_1, u_2) \in E_s$, and $(u_2, u) \in E_d$, and setting $\ell_{u_1 u_2} = 1$. This construction allows alternate paths in the model to traverse through foldbacks and, by assigning a positive length, encourages their inclusion in the solution based on the objective function.

**Connectivity enforcement cycle extraction.** When CEc aims to extract a cycle (to avoid multiple disconnected cycles), it connects artificial source ($s$) and sink ($t$) nodes to all nodes in the graph. To extract exactly one cycle, CEc finds a path from $s$ to $t$, with $s$ and $t$ connected to the same node in the solution. To ensure feasibility with respect to the alternating edge constraints, $s$ is connected to other nodes via discordant edges, while $t$ is connected via sequence edges. The copy number assigned to each of these newly added edges equals the maximum copy number of the edges originally adjacent to that node. The length of the sequence edge connecting $t$ is set to 1 - positive, but short - so that it encourages selection in the solution.

**Extract paths from graph.** The CE(c) method is an iterative framework that, in each iteration, takes as input an amplicon graph with copy number annotated edges. It formulates and solves a mixed-integer linear programming (MILP) problem to identify a subset of edges, each with an assigned copy number, that can form a cycle. After each iteration, the graph is updated by subtracting the copy numbers of the selected edges from their corresponding values in the graph. The updated graph is then used as input for the next iteration to extract additional cycles. Although CE/CEc can also extract paths, it prioritizes the detection of cycles. Once no cycles remain, the algorithm proceeds to extract paths.

If CE/CEc aims to extract a path after all cycles have been extracted, it introduces artificial source ($s$) and sink ($t$) nodes. Both nodes are connected to all other nodes in the graph via discordant edges, and each new edge is assigned a copy number equal to the maximum copy number of the edges originally adjacent to that node.

## S1.5 Cell lines used in this paper

For building the amplicon graph, we used the following: AmpliconArchitect (AA) version 1.5.r1; CoRAL version 2.2.0.

## S1.6 Metaphase FISH

Half a million PC3-DM5 cells were seeded on a 6mm dish followed by a 4-hour KaryoMAX Colcemid (Gibco) treatment at 100ng mL$^{-1}$ to arrest cells into metaphase. Cells were then trypsinized, washed in 1X PBS, followed by a 20-min 0.075M KCl hypotonic buffer incubation at 37°C. Freshly prepared Carnoy's fixative (3:1 methanol:glacial acetic acid) was added to the cells, fully resuspended by pipetting and spun down. Two additional washes in Carnoy's fixative were performed,

bioRxiv preprint doi: https://doi.org/10.64898/2026.03.10.710955; this version posted April 15, 2026. The copyright holder for this preprint (which was not certified by peer review) is the author/funder, who has granted bioRxiv a license to display the preprint in perpetuity. It is made available under a CC-BY 4.0 International license.

| Cell line | Source | Genes | ONT | Illumina |
|---|---|---|---|---|
| TR14 | Hung 2021[26] | *MDM2* | ✓ | ✓ |
| TR14 | Hung 2021[26] | *MYCN, CDK4* | ✓ | ✓ |
| TR14 | Hung 2021[26] | *ODC1* | ✓ | |
| TR14 | Hung 2021[26] | *SMC6* | ✓ | |
| HK296 | Turner 2017[1] | *MDM4* | | ✓ |
| HK296 | Turner 2017[1] | *EGFR* | | ✓ |
| GBM39 | Turner 2017[1] | *EGFR* | ✓ | ✓ |
| GBM39_mono | Zhu 2024[35] | *EGFR* | ✓ | |
| SF295 | Turner 2017[1] | *CCND1* | | ✓ |
| H460 | Turner 2017[1] | MYC | | ✓ |
| MB411FH | Turner 2017[1] | *MYC* | | ✓ |
| CA718 | Turner 2017[1] | *MDM2, MYCN, PDGFRA* | | ✓ |
| CHP-212 | Ghandi 2019[48] | *MYCN* | ✓ | ✓ |
| UACC1598 | Luebeck 2024[31] | *MYCN* | | ✓ |
| CCFSTTG1 | Luebeck 2024[31] | *CDK6, MDM2* | | ✓ |
| HL-60 | Turner 2017[1] | *MYC* | | ✓ |
| NCIH2170 | Dehkordi 2025[49] | *MYC* | ✓ | ✓ |
| MB002 | Turner 2017[1] | *MYC* | | ✓ |
| HK301 | Turner 2017[1] | *EGFR* | | ✓ |
| H524 | Luebeck 2024[31] | *MYC* | | ✓ |
| NCIH69 | Ghandi 2019[48] | *MYCN* | | ✓ |
| HK359 | Turner 2017[1] | *EGFR* | | ✓ |
| GBM6 | Turner 2017[1] | *EGFR* | | ✓ |
| DMS273 | Ghandi 2019[48] | *MYC* | | ✓ |
| DMS273 | Ghandi 2019[48] | *MYCL, ARNT* | | ✓ |
| COLO320DM | Zhu 2024[35] | *MYC* | ✓ | ✓ |
| COLO320DM_Hung2021 | Hung 2021[26] | *MYC* | ✓ | |
| H716 | Jia 2020[50] | *MYC, FGFR2* | | ✓ |
| SCLC21H | Ghandi 2019[48] | *MYC* | | ✓ |
| PC3 | Turner 2017[1] | *MYC* | ✓ | ✓ |
| SNU16 | Hung 2022[38] | *MYC, FGFR2* | ✓ | |

Supplementary Table S2: **Amplicons used for testing cycle extraction.** Each row describes a unique amplicon defined by its amplicon graph.

and the cell pellet was resuspended in Carnoy's fixative. Cells were dropped onto a humidified glass slide and allowed to air-dry. The slide was briefly equilibrated in 2X SSC buffer, followed by dehydration in ascending ethanol concentrations of 70%, 85% and 100% for 2 mins each. DNA FISH probes (Empire Genomics) were diluted at 1:10 in hybridization buffer. The following FISH probes were used to detect *MYC* (RP11-440N18), *SAMD12* (RP11-671M23) and *FER1L6* (RP11-1112G17). The probe mixture was added to the sample and covered with a 22x22 coverslip. The sample was subjected to a 3-min heat denaturation at 75°C, followed by an overnight hybridization at 37°C in a humidified slide moat. The next day, the coverslip was carefully removed from the glass slide and the sample was washed in 0.4X SSC for 2 mins, followed by another 2-min wash with 2X SSC containing 0.1% Tween 20. The slide was incubated with 2X SSC containing DAPI (50 ng $mL^{-1}$) for another 10 mins to stain DNA content. Followed by a brief ddH2O wash, the sample was mounted with ProLong Diamond Antifade and air-dried. Images were acquired on a Leica DMi8 widefield microscope with a 63x oil objective.
